# (Bio)isosteres of *ortho*- and *meta*-substituted benzenes

**DOI:** 10.3762/bjoc.20.78

**Published:** 2024-04-19

**Authors:** H Erik Diepers, Johannes C L Walker

**Affiliations:** 1 Institut für Organische und Biomolekulare Chemie, Georg-August-Universität Göttingen, Tammannstraße 2, 37077 Göttingen, Germany

**Keywords:** bioisosteres, drug discovery, *meta*-benzene, *ortho*-benzene

## Abstract

Saturated bioisosteres of substituted benzenes offer opportunities to fine-tune the properties of drug candidates in development. Bioisosteres of *para*-benzenes, such as those based on bicyclo[1.1.1]pentane, are now very common and can be used to increase aqueous solubility and improve metabolic stability, among other benefits. Bioisosteres of *ortho*- and *meta*-benzenes were for a long time severely underdeveloped by comparison. This has begun to change in recent years, with a number of potential systems being reported that can act as bioisosteres for these important fragments. In this review, we will discuss these recent developments, summarizing the synthetic approaches to the different bioisosteres as well as the impact they have on the physiochemical and biological properties of pharmaceuticals and agrochemicals.

## Introduction

**Figure 1:**
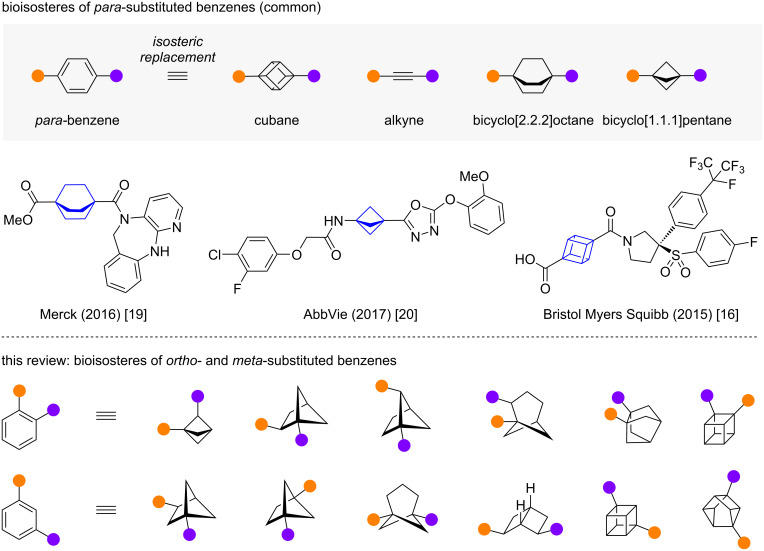
Scaffolds commonly reported as bioisosteric replacements of *para*-substituted benzene and examples patented by Merck [19], AbbVie [20] and Bristol-Myers Squibb [16].

The logical and iterative modification of the structure of a drug candidate is a critical part of the early drug discovery process. In many cases, the primary aim is the enhancement of biological activity, but in others, modulation of other critical properties such as aqueous solubility, metabolic stability, polarity, or lipophilicity is the target. In the latter cases, there is often a desire to retain otherwise potent biological activity and here the application of bioisosteric fragments can play a leading role. Bioisosteres are structurally distinct from the groups they replace, leading to changes in the physiochemical properties of the overall compound, but allow for retention of the biological activity, if not an improvement [1]. Often-used bioisosteres include the tetrazole group for a carboxylic acid [2–5] and fluorine atoms in place of hydrogens [6–7]. The inclusion of fluorine can alter the polarity of a molecule and can also be used to prevent epimerisation, as seen in fluorothalidomide [8]. Similarly, the reduction in the number of aromatic groups and increase in the level of saturation have risen to prominence as a way of improving low aqueous solubility, metabolic instability, or low lipophilicity in drug candidates that contain a high number of these functionalities [9–10]. The direct replacement of substituted benzenes in drug candidates with saturated benzene bioisosteres is a popular approach to this task [11–14]. Substitution of a mono-substituted benzene (a phenyl group) is relatively straightforward [11,13,15]. More particular attention must be paid to benzenes that have more than one substituent; for example, the *ortho*-, *meta*-, or *para*- relative substitution of a disubstituted benzene should ideally be replicated in the saturated bioisostere to ensure ligand–protein binding is conserved through the bioisosteric swap. Bioisosteres of *para*-substituted benzenes are by now relatively well established. Cubanes [16–17], alkynes [18], and bicyclo[2.2.2]octanes [19] can all replicate the linear geometry of the *para*-substituents, but arguably the most well-investigated *para*-benzene bioisostere is bicyclo[1.1.1]pentane (BCP) [20–23] (Figure 1). The straightforward synthesis of a wide-range of disubstituted BCPs from [1.1.1]propellane allows them to be easily integrated into drug discovery programmes. The case of Darapladib is one well-known example of a productive *para*-benzene-to-BCP bioisosteric switch [24]. By comparison, the number of bioisosteres of *ortho*- and *meta*-benzenes was for a long time low [14], but recent years have seen a surge in interest in developing such systems. In these cases, replication of the 60° and 120° bond angles of *ortho*- and *meta*-benzenes is a key challenge. This review will summarise the progress made so far towards the development of these important bioisosteres. We will highlight both the synthetic routes used to access the proposed bioisosteric scaffolds, as well as any subsequent derivatization thereof which help expand their utility. Our aim is to provide an overview of the types of scaffold that can be prepared with each method, enabling practitioners to quickly find the bioisostere and method that best suits their requirements. Physiochemical and biological data will also be discussed where available.

## Review

### Definitions and parameters for determination of (bio)isosterism

We will distinguish between geometric isosteres and bioisosteres. Scaffolds suggested for the replacement of *ortho*- or *meta*-benzenes shall be defined as a geometrical isostere if they exhibit similar geometric properties to the parent compound. A scaffold shall be defined as a bioisostere only if both its geometrical properties and its biological activity are similar to the parent compound. For simplicity, geometric isosteres will be simply referred to as isosteres. More densely substituted scaffolds will be briefly discussed at the end of the review.

Geometrical equivalence between disubstituted benzenes and scaffolds will be quantified in exit vector analyses [25]. Here, the geometric relationship between the two substituents is measured and compared to those of the parent benzene.

To compare physicochemical properties, the following indicators will generally be used:

1. distribution coefficient (logD, desired range: 1–3 [26]) or partition coefficient (logP) as a measure of compound polarity,
2. aqueous solubility (desired value: >200 μM [26]), and
3. intrinsic clearance rate (CL_int_, lower indicates higher metabolic stability) as a measure of metabolic stability. Additional properties will be discussed if comparative values have been reported.

### Isosteres of *ortho*-substituted benzenes

#### 1,2-Disubstituted bicyclo[1.1.1]pentanes

**Figure 2:**
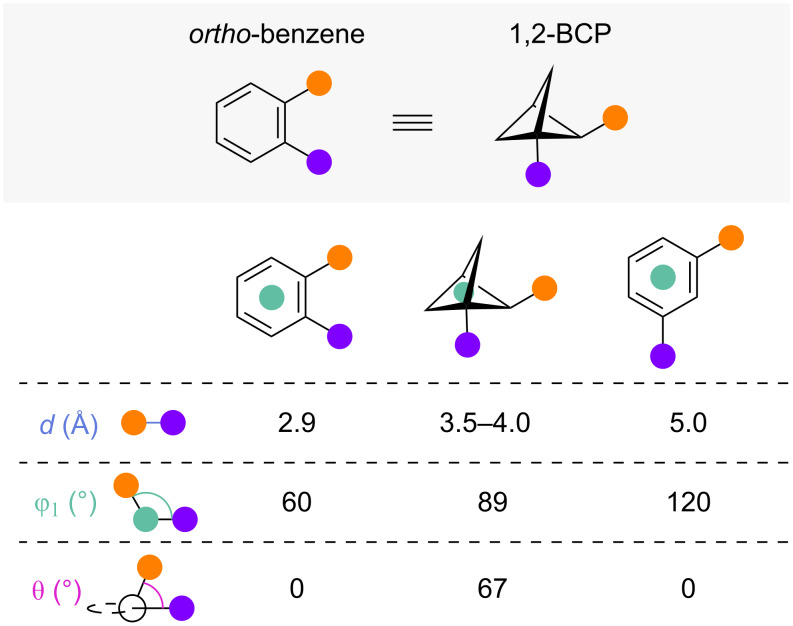
1,2-BCPs as isosteres for *ortho-*and *meta-*substituted benzenes: comparison of reported exit vector parameters [26–27].

Among the first suggested isosteres for *ortho*-substituted benzenes were 1,2-disubstituted bicyclo[1.1.1]pentanes (1,2-BCPs). The exit vector analysis of 1,2-BCPs and *ortho-* or *meta*-substituted benzenes performed by Baran, Collins and co-workers and Anderson and co-workers showed that the substituent distance *d* of 3.5–4.0 Å and substituent angle φ_1_ of 89° inhabit a chemical space between *ortho*- and *meta*-substituted benzene (Figure 2) [26–27]. The 67° dihedral angle θ between the substituents differs significantly from both compounds.

**Scheme 1:**
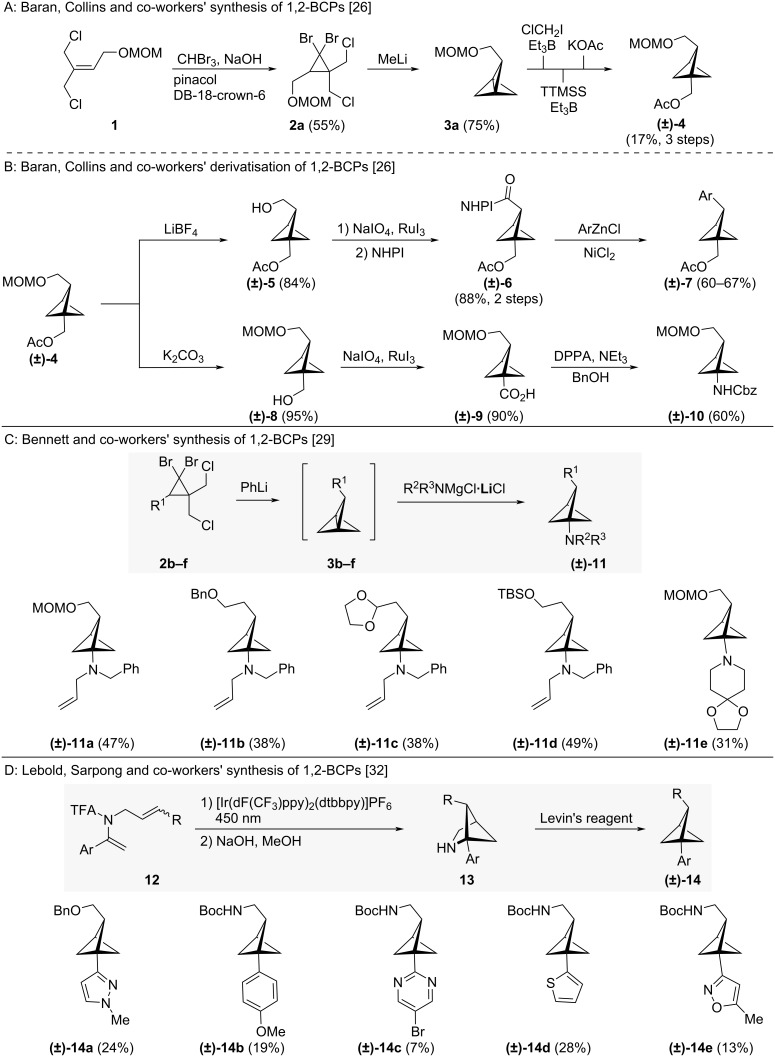
1,2-Disubstituted bicyclo[1.1.1]pentanes as isosteres of *ortho-*substituted benzenes. A: Baran, Collins and co-workers’ synthesis of [1.1.1]propellane **3a** and transformation to BCP **(±)-4** [26]. B: Reactions of BCP **(±)-4** to differently-substituted BCPs [26]. C: Synthesis of 1-dialkylamino-1,2-BCPs by Bennett and co-workers [29]. D: Lebold, Sarpong and co-workers’ synthesis of 1,2-disubstituted BCPs [32]. DB = Dibenzo, TTMSS = tris(trimethylsilyl)silane, NHPI = *N*-hydroxyphthalimide, DPPA = diphenylphosphoryl azide, Levin’s reagent = *N*-(benzyloxy)-1-[4-(trifluoromethyl)phenyl]formamido 2,2-dimethylpropanoate.

The first investigation of 1,2-BCPs as an isosteric replacement for *ortho*-benzene was reported by Baran, Collins and co-workers [26]. They prepared a wide variety of 1,2-BCPs from a common 2-substituted [1.1.1]propellane building block, propellane **3a** (Scheme 1A). Propellane **3a** was itself accessible in good yields from allyl chloride **1** using a route based on that reported by Schlüter [28]. Bifunctional 1,2-BCP **(±)-4** bearing orthogonally protected alcohol functionalities was obtained from **3a** through a three-step sequence of strain-release radical ring-opening with iodochloromethane, deiodination at the bridgehead position, and nucleophilic substitution at the alkyl chloride.

From 1,2-BCP **(±)-4**, a variety of 1,2-BCPs were prepared through basic chemical transformations (Scheme 1B) [26]. Selective deprotection gave access to free alcohol-containing 1,2-BCPs **(±)-5** and **(±)-8**. Oxidation and esterification of alcohol **(±)-5** gave redox active ester **(±)-6**, which was itself shown to be a suitable substrate for nickel-catalysed decarboxylative cross coupling reactions to aryl-substituted BCPs **(±)-7**. Oxidation of alcohol **(±)-8** gave acid **(±)-9** which yielded amine **(±)-10** after a Curtius rearrangement.

Bennet and co-workers also reported the synthesis of 1-amino-1,2-BCPs **(±)-11a–e** via a similar strategy (Scheme 1C) [29]. They were able to prepare differently-substituted [1.1.1]propellanes **3b–f** and subject these to strain-release amination reactions. The synthesis was shown to tolerate typical alcohol and aldehyde protecting groups.

Recently, Lebold, Sarpong, and co-workers showed that 1,2-BCPs **(±)-14a–e** are also accessible from 1,5-disubstituted 2-azabicyclo[2.1.1]hexanes **13** (2-aza-1,5-BCHs) through a skeletal editing strategy utilising commercially available Levin’s reagent [30–31] (Scheme 1D) [32]. The synthesis of the corresponding 2-aza-1,5-BCHs **13** was achieved by an intramolecular photochemical [2 + 2] cycloaddition. They were able to employ this synthetic route to synthesise a variety of 1,2-BCPs **(±)-14** bearing a protected amine in the bridge position.

**Scheme 2:**
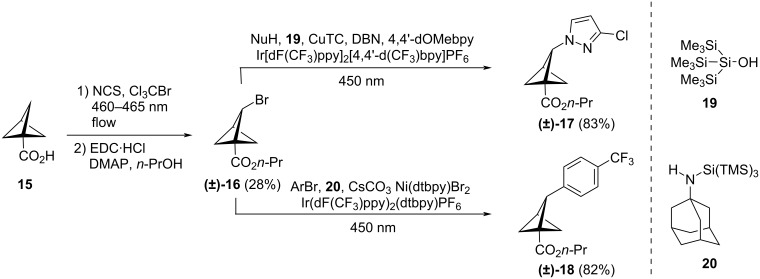
Synthesis of 1,2-BCPs from BCP **15** by bridge C–H bromination as reported by MacMillan and co-workers [33]. DBN = 1,5-Diazabicyclo[4.3.0]non-5-ene.

In early 2023, MacMillan and co-workers reported a method for the selective bridge bromination of BCPs, giving access to brominated 1,2-BCP **(±)-16** (Scheme 2) [33]. By exploiting the homolytic cleavage of the C–Br bond using in situ*-*generated silyl radicals, they were then able to harness the installed bromide functionality in metallophotoredox coupling reactions to deliver nitrogen*-* and aryl-functionalised BCPs such as **(±)-17** and **(±)-18**.

**Figure 3:**
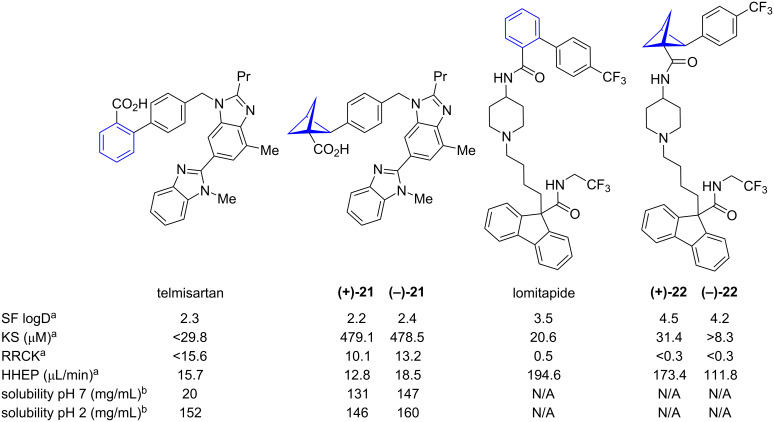
Comparative physicochemical data of telmisartan, lomitapide and their BCP isosteres [26,33]. Shake flask distribution coefficient (SF logD), kinetic aqueous solubility (KS), Ralph Russ canine kidney (RRCK), human hepatocyte stability (HHEP). ^a^Reported by Baran, Collins and co-workers, ^b^Reported by MacMillan and co-workers.

In the reports of Baran, Collins and co-workers and of MacMillan and co-workers, isosteres of compounds including telmisartan (isostere = BCP **21**) and lomitapide (isostere = BCP **22**) were prepared and their physicochemical properties were compared to those of the parent compounds (Figure 3) [26,33]. The reported distribution coefficients (logD) of enantiomeric 1,2-BCPs **(+)-21** and **(−)-21** are very similar to telmisartan, while the distribution coefficients of the lomitapide isosteres **(+)-22** and **(−)-22** are significantly increased compared with the parent compound. The observed aqueous solubilities of BCPs **(+)-21** and **(−)-21** were much higher than that of telmisartan. For the lomitapide isosteres **(+)-22** and **(−)-22**, the solubility was different for each enantiomer; while 1,2-BCP **(+)-22** showed increased solubility, the solubility of 1,2-BCP **(–)-22** was decreased compared to lomitapide. Cell permeability, as determined by Ralph Russ canine kidney essay (RRCK), was reduced in all 1,2-BCP isosteres compared to the parent compounds. Metabolic stability, as determined by human hepatocyte stability (HHEP), was generally improved by isosteric replacement, the single exception being telmisartan isostere **(−)-21**.

#### 1,2-Disubstituted bicyclo[2.1.1]hexanes

**Figure 4:**
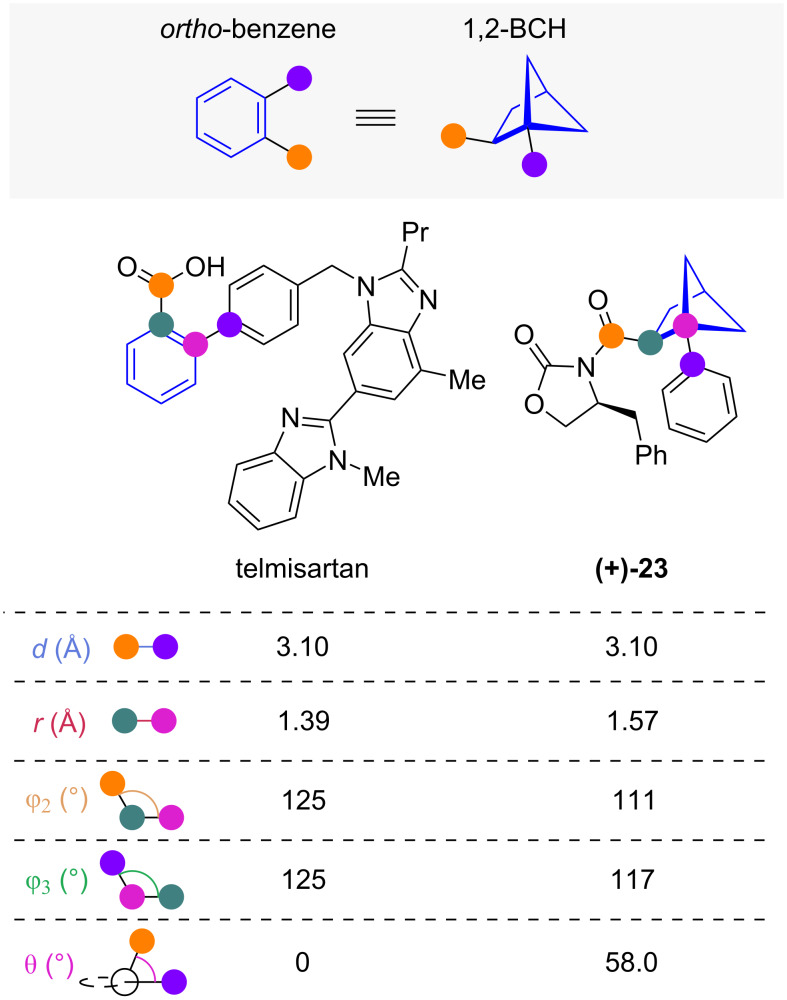
1,2-Disubstituted bicyclo[2.1.1]hexanes as isosteres of *ortho*-benzenes: Exit vector parameters of telmisartan and 1,2-BCH **(+)-23** obtained through X-ray crystallography reported by Walker and co-workers [34].

1,2-Disubstituted bicyclo[2.1.1]hexanes (1,2-BCHs) have been proposed as isosteres for *ortho-*substituted benzenes [14,34–35]. Comparison of exit vector parameters of 1,2-BCH **(+)-23** and *ortho-*benzene telmisartan has been reported by Walker and co-workers (Figure 4) [34]. They found that both the substituent distance *d* and scaffold carbon distance *r* of 1,2-BCH **(+)-23** closely resemble the ones found in telmisartan. While this agreement extends to the substituent scaffold angles φ_2_ and φ_3_, the 58° dihedral angle of 1,2-BCH **(+)-23** is significantly larger than the 0° found in telmisartan. An exit vector analysis of 1,2-BCHs and *ortho*-benzene independently reported by Mykhailiuk and co-workers confirms these geometrical trends [36].

**Scheme 3:**
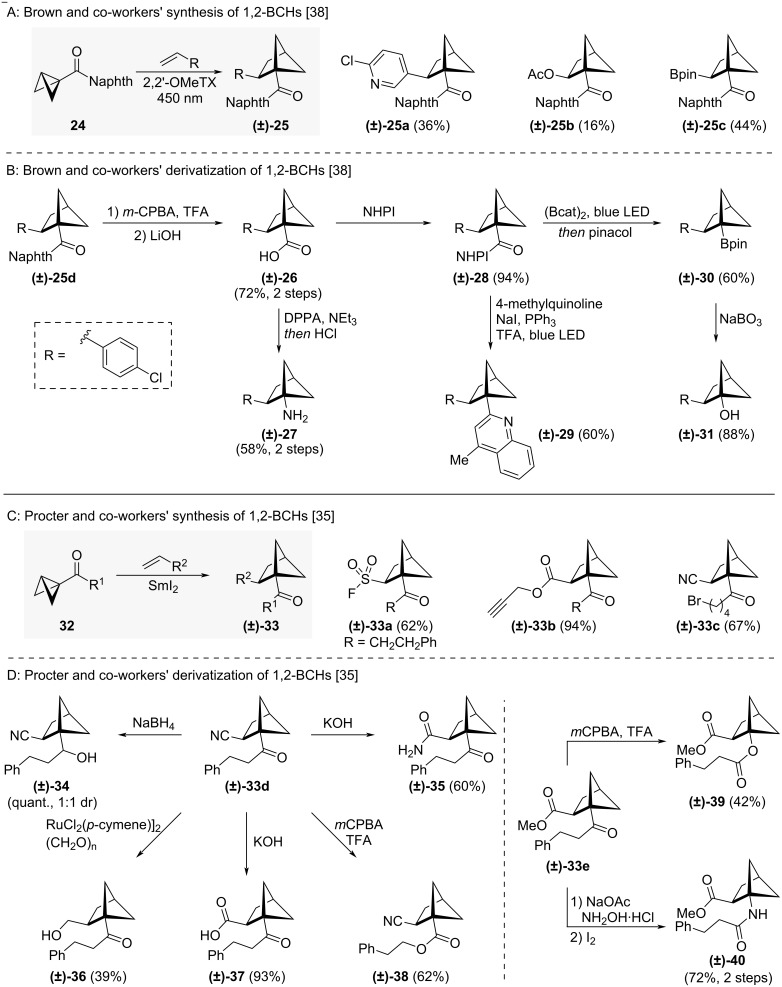
Synthesis of 1,2-disubstituted bicyclo[2.1.1]hexanes via alkene insertion into bicyclo[1.1.0]butanes. A: Brown and co-workers’ synthesis and representative substrate scope of 1,2-disubstituted BCHs [38]. B: Brown and co-workers’ synthesis of bifunctional 1,2-BCHs [38]. C: Procter and co-workers’ synthesis of 1,2-disubstituted BCHs [35]. D: Procter and co-workers’ derivatization of BCHs [35]. 2,2’-OMeTX = 2,7-dimethoxythioxanthone, cat = catechol, pin = pinacol, NHPI = *N*-hydroxyphthalimide, DPPA = diphenylphosphoryl azide, *m*CPBA = *meta*-chloroperbenzoic acid, TFA = trifluoroacetic acid.

For a long time, routes to 1,2-BCHs were rare, but a number of practical approaches have been disclosed in recent years. Many of these utilise the ability of bicyclo[1.1.0]butanes (BCBs) to undergo [2σ-2π]-type cycloadditions with alkene reaction partners [37]. Brown and co-workers used this approach to synthesise a variety of 1,2-BCHs in moderate to good yields, employing 2,7-dimethoxythioxanthone (2,2’-OMeTX) as a triplet sensitizer for BCB excitation (Scheme 3A) [38]. Starting from BCB **24**, alkenes including styrene derivatives, enol ethers, and vinyl boronates could be incorporated to give 1,2-BCHs **(±)-25a–d**. Brown and co-workers also reported the modification of these 1,2-BCHs to increase the number of derivatives accessible using this approach (Scheme 3B) [38]. Transformation of the naphthyl ketone moiety of BCH **(±)-25d** by Baeyer–Villiger oxidation followed by hydrolysis gave carboxylic acid **(±)-26**. Through Curtius rearrangement, amine **(±)-27** was then accessible in one additional step. Formation of redox active ester **(±)-28** from acid **(±)-26** allowed photochemical Minisci reaction to 1,2-BCH **(±)-29** and borylation to boronic ester **(±)-30**. Synthesis of phenol isostere **(±)-31** was possible through oxidation of boronic ester **(±)-30**.

In a related strategy, Procter and co-workers prepared 1,2-BCHs **(±)-33a–e** from BCBs **32** via a SmI_2_-catalysed radical relay alkene insertion (Scheme 3C) [35]. This approach relied on single-electron reduction of the ketone moiety and ring-expansion from the ketyl radical anion. Electron-deficient alkenes including acrylates, vinyl sulfones, and acrylamides could all be incorporated and the number of accessible bifunctional 1,2-BCHs was increased further by chemical transformation (Scheme 3D) [35]. Among the reported transformations were reduction of the ketone (to **(±)-34**), hydrolysis of the nitrile group (to **(±)-35** and **(±)-37**), Baeyer–Villiger oxidation (to **(±)-38** and **(±)-39**), reduction of the nitrile group (to **(±)-36**) and Beckman rearrangement (to **(±)-40**).

A mechanistically related synthesis of 1,2-BCHs was published recently by Wang and co-workers (not shown) [39]. They employed a catalytic system of B_2_cat_2_ and 4-phenylpyridine to form pyridine-boryl radicals which initiated ring expansion. The method was shown to be similarly tolerable of functional groups as Procter’s synthesis.

**Scheme 4:**
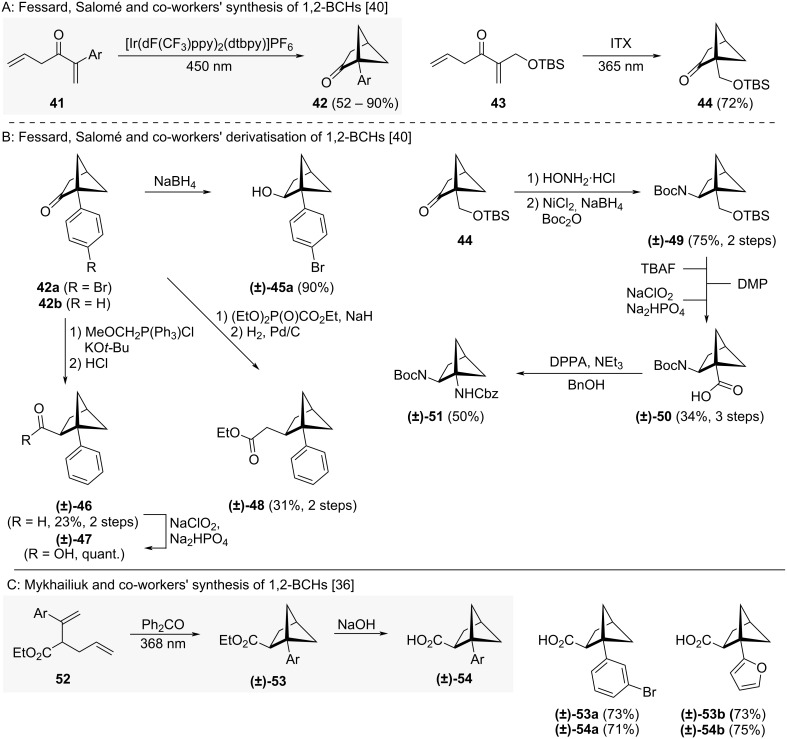
Synthesis of 1,2-disubstituted bicyclo[2.1.1]hexanes via intramolecular crossed [2 + 2] cycloaddition. A: Fessard, Salomé and co-workers’ synthesis of 1,2-BCHs as precursors for *ortho*-substituted benzene isosteres [40]. B: Fessard, Salomé and co-workers’ derivatization of 1,2-BCHs [40]. C: Mykhailiuk and co-workers’ synthesis of 1,2-BCHs as *ortho*-substituted benzene isosteres [36]. TBS = *tert*-Butyldimethylsilyl, ITX = 2-isopropylthioxanthone, DMP = Dess–Martin periodinane, DPPA = diphenylphosphoryl azide.

Intramolecular crossed [2 + 2] cycloadditions offer an alternative approach to 1,2-disubstituted BCHs. Fessard, Salomé and co-workers used this approach to synthesise a small collection of 2-oxo-BCHs including **42** and **44** (Scheme 4A) [40]. These could then be used as precursors for *ortho*-benzene isosteric 1,2-BCHs (Scheme 4B) [40]. Carbonyl reduction of **42a** yielded alcohol **(±)-45a**. From **42b**, Wittig homologation and hydrolysis led to aldehyde **(±)-46** which could then be oxidised to acid **(±)-47** using a Pinnick oxidation. BCH **42b** also led to ester **(±)-48** via a Horner–Wadsworth–Emmons reaction followed by hydrogenation of the formed alkene. 1,2-BCH **44** could be turned into amine **(±)-49** by oxime formation and reduction. Deprotection of the alcohol followed by two-step oxidation to the carboxylic acid yielded the non-natural β-amino acid **(±)-50**, which could then be transformed into diamine **(±)-51** by Curtius rearrangement.

Recently, Mykhailiuk and co-workers also reported the synthesis of 1,2-BCHs using an intramolecular crossed [2 + 2] cycloaddition strategy (Scheme 4C) [36]. They were able to avoid purification by column chromatography by transformation of BCH ester **(±)-53** into BCH acid **(±)-54**, allowing isolation of pure 1,2-BCH **(±)-54** by crystallisation. Through their synthesis they were able to access both aryl (**(±)-54a**) and heteroaryl (**(±)-54b**) substituted 1,2-BCHs.

As an alternative synthesis of 1,2-BCHs, and importantly also enantioenriched examples, Qin and co-workers developed an intramolecular coupling of cyclobutane-tethered sulfonylhydrazones and boronic esters (not shown) [41]. They also employed their method for the synthesis of 1,2,3-trisubsituted BCPs and a number of other bridged bicyclic scaffolds.

**Figure 5:**
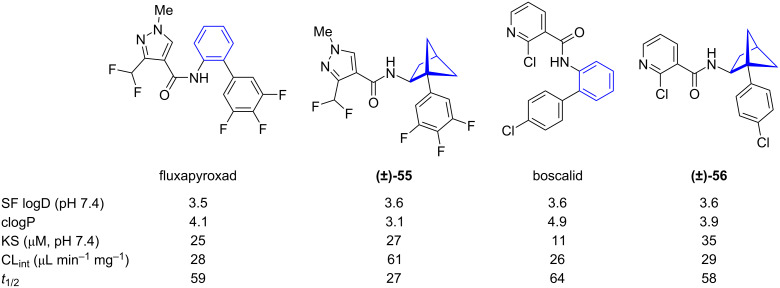
Comparison of physicochemical data of fluxapyroxad and boscalid and their 1,2-BCH bioisosteres [36]. Shake flask distribution coefficient (SF logD), calculated partition coefficient (clogP), kinetic solubility in saline (KS), intrinsic clearance in human liver microsomes (CL_int_), metabolic decomposition half-time (*t*_1/2_).

Comparison of physicochemical data of 1,2-BCHs and *ortho*-benzenes was reported by Mykhailiuk and co-workers (Figure 5) [36]. Lipophilicity of 1,2-BCHs was measured by experimental distribution coefficient (SF logD) and calculated partition coefficient (clogP). This analysis showed while the calculated partition coefficient was reduced by bioisosteric replacement of *ortho*-benzene the experimental distribution coefficient did not change significantly. However, while aqueous solubility increased by bioisosteric replacement of *ortho*-benzene with 1,2-BCHs, the metabolic stability as determined by intrinsic clearance rate in human liver microsomes (Cl_int_) and half-time of metabolic decomposition (*t*_1/2_) decreased.

**Figure 6:**
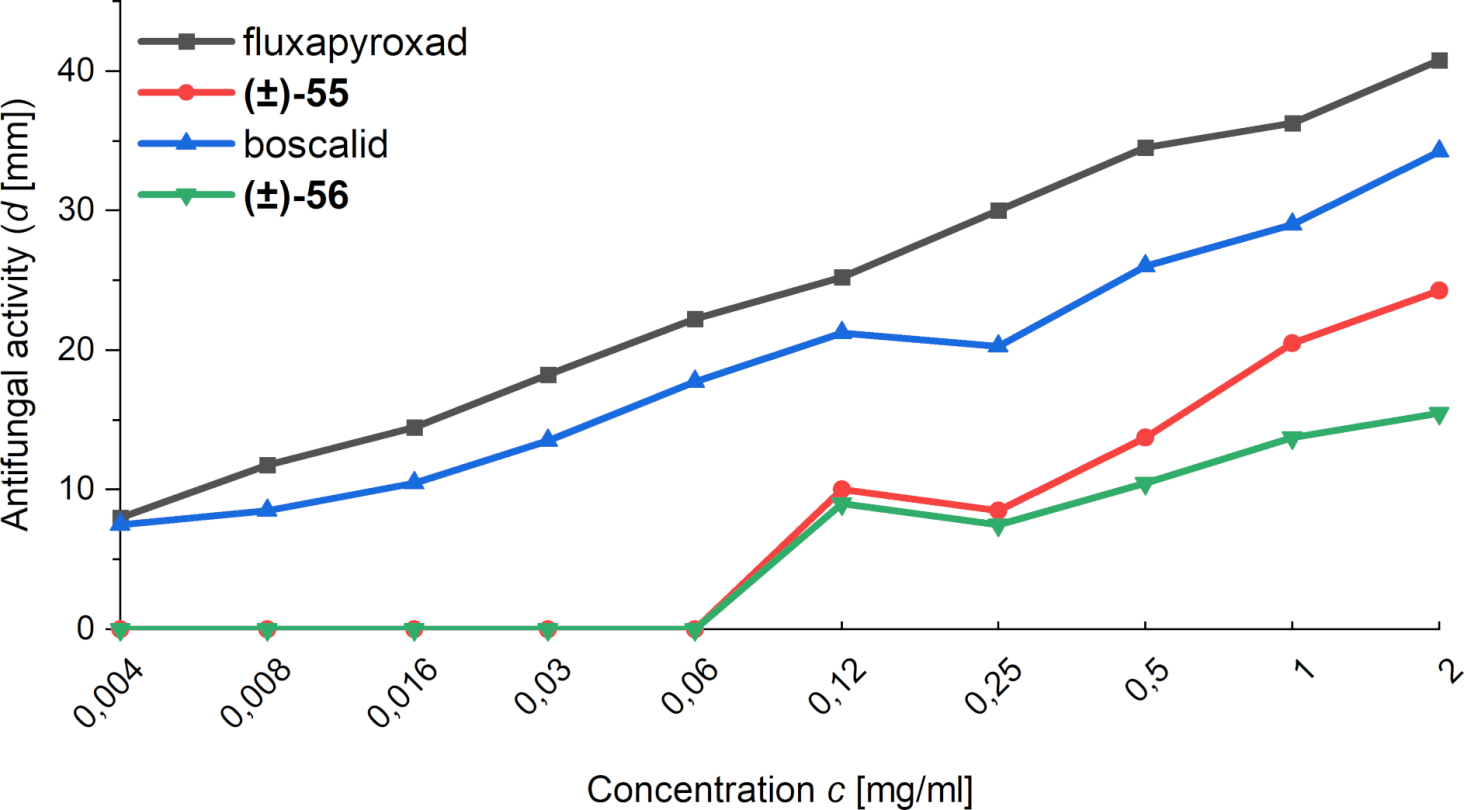
Antifungal activity of fluxapyroxad, its 1,5-BCH bioisostere **(±)-55**, boscalid and its bioisostere 1,5-BCH **(±)-56** against *Aspergillus niger* in dependence on the concentration of the fungicide [36].

Mykhailiuk and co-workers also reported the antifungal activity of fluxapyroxad and boscalid biosisosteres **(±)-55** and **(±)-56** (Figure 6) [36]. The comparison of the antifungal activity over a wide range of concentrations showed that while biological activity is preserved upon incorporation of the 1,2-BCH scaffold, it is significantly reduced. This was also the case for the fungicide bixafen and its 1,2-BCH-based bioisostere, as reported in the same publication (not shown).

#### 1,5-Disubstituted bicyclo[2.1.1]hexanes

**Figure 7:**
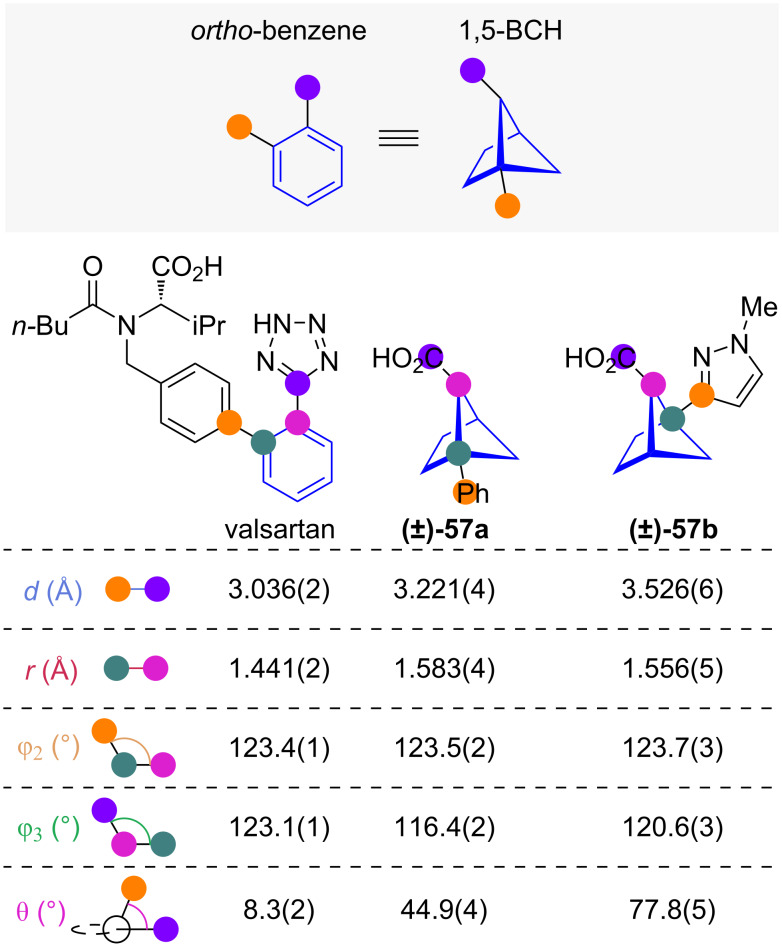
1,5-Disubstituted bicyclo[2.1.1]hexanes as isosteres of *ortho*-substituted benzenes. Comparison of exit vector parameters for *ortho*-benzene (valsartan) and 1,5-BCH isosteres (**57a**, **57b**) obtained from X-ray structures as reported by Mykhailiuk and co-workers [42].

The isomeric 1,5-disubstituted bicyclo[2.1.1]hexanes (1,5-BCHs) have also been suggested as *ortho*-benzene isosteres [42]. Exit vector analysis reveals that the distance between the *ortho* substituents *d* is slightly larger in 1,5-BCH than in *ortho*-benzene (Figure 7) [42]. The substituent–arene angles φ_2_ and φ_3_ are similar, but the dihedral angle θ between substituents is necessarily larger in 1,5-BCHs than the near 0° angle for *ortho*-benzenes.

**Scheme 5:**
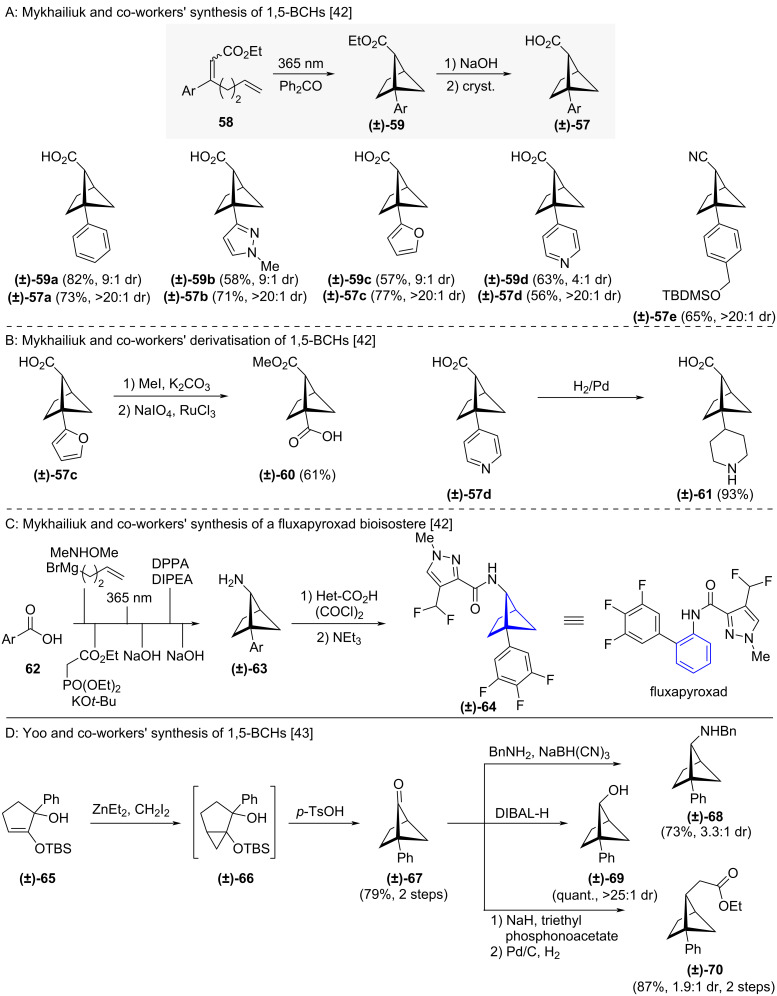
Synthesis of 1,5-disubstituted bicyclo[2.1.1]hexanes as isosteres of *ortho*-benzenes via intramolecular [2 + 2] cycloaddition A: Reaction sequence developed by Mykhailiuk and co-workers for the synthesis of 1,5-BCHs and selected examples of the reported substrate scope [42]. B: Transformations of 1,5-BCHs to bifunctional scaffolds reported by Mykhailiuk and co-workers [42]. C: Application of the Mykhailiuk and co-workers’ synthesis for the synthesis of a fluxapyroxad bioisostere [42]. D: Yoo and co-workers’ synthesis of 1,5-BCHs [43].

Mykhailiuk and co-workers adopted a similar intramolecular [2 + 2] cycloaddition using benzophenone as a triplet sensitizer to access 1,5-BCHs **(±)-59a–e** as racemic mixtures of *endo*:*exo* diastereomers (Scheme 5A) [42]. Basic hydrolysis of the ester moiety followed by recrystallisation gave the diasteromerically pure acid-substituted *endo*-1,5-BCHs **(±)-57a–e**. Substrate modification was also possible, with oxidative transformation of a bridgehead furan leading to 1,5-BCH acid **(±)-60** and pyridine reduction to secondary amine **(±)-61** (Scheme 5B) [42]. Furthermore, 1,5-BCH **(±)-64** was synthesised as a bioisostere of the fungicide fluxapyroxad from amine **(±)-63**, which was itself accessed via Curtius rearrangement of the corresponding 1,5-BCH (Scheme 5C) [42]. In 2023, Yoo and co-workers reported the synthesis of 1,5-BCHs by derivatization of 5-oxo-BCHs **(±)-67** (Scheme 5D) [43]. They accessed the 5-oxo-BCHs by Simmons–Smith cyclopropanation [44] (to **(±)-66**) of α-hydroxy silyl enol ethers **(±)-65** followed by an acid-catalysed pinacol rearrangement to **(±)-67**. As exemplary derivatizations of 5-oxo-BCH **(±)-67**, 1,5-BCHs **(±)-68, (±)-69** and **(±)-70** were accessed by reductive amination, ketone reduction, and Horner–Wadsworth–Emmons olefination with subsequent reduction of the olefin motif respectively. In contrast to the approach of Mykhailiuk, this route yields the *exo*-1,5-BCHs as the major diastereomer.

**Figure 8:**
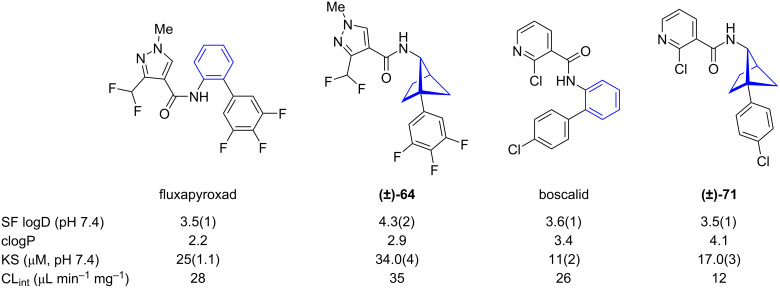
Comparison of physicochemical data of fluxapyroxad and boscalid and their 1,5-BCH bioisosteres [45]. Shake flask distribution coefficient (SF logD), calculated partition coefficient (clogP), kinetic solubility in saline (KS), intrinsic clearance in human liver microsomes (CL_int_).

1,5-BCH **(±)-64** and 1,5-BCH **(±)-71** were prepared as bioisosteres of the fungicides fluxapyroxad and boscalid, respectively [45]. Comparison of physicochemical data showed that the experimentally determined distribution coefficient (logD) and calculated partition coefficient (clogP) are slightly higher for the bioisosteres and the aqueous solubility at pH 7.4 was also increased (Figure 8) [45]. Fluxapyroxad bioisostere **(±)-64** had a higher clearance rate in human liver microsomes (CL_int_) than fluxapyroxad (lower metabolic stability) but boscalid bioisostere **(±)-71** had a lower CL_int_ and was more metabolically stable than boscalid.

**Figure 9:**
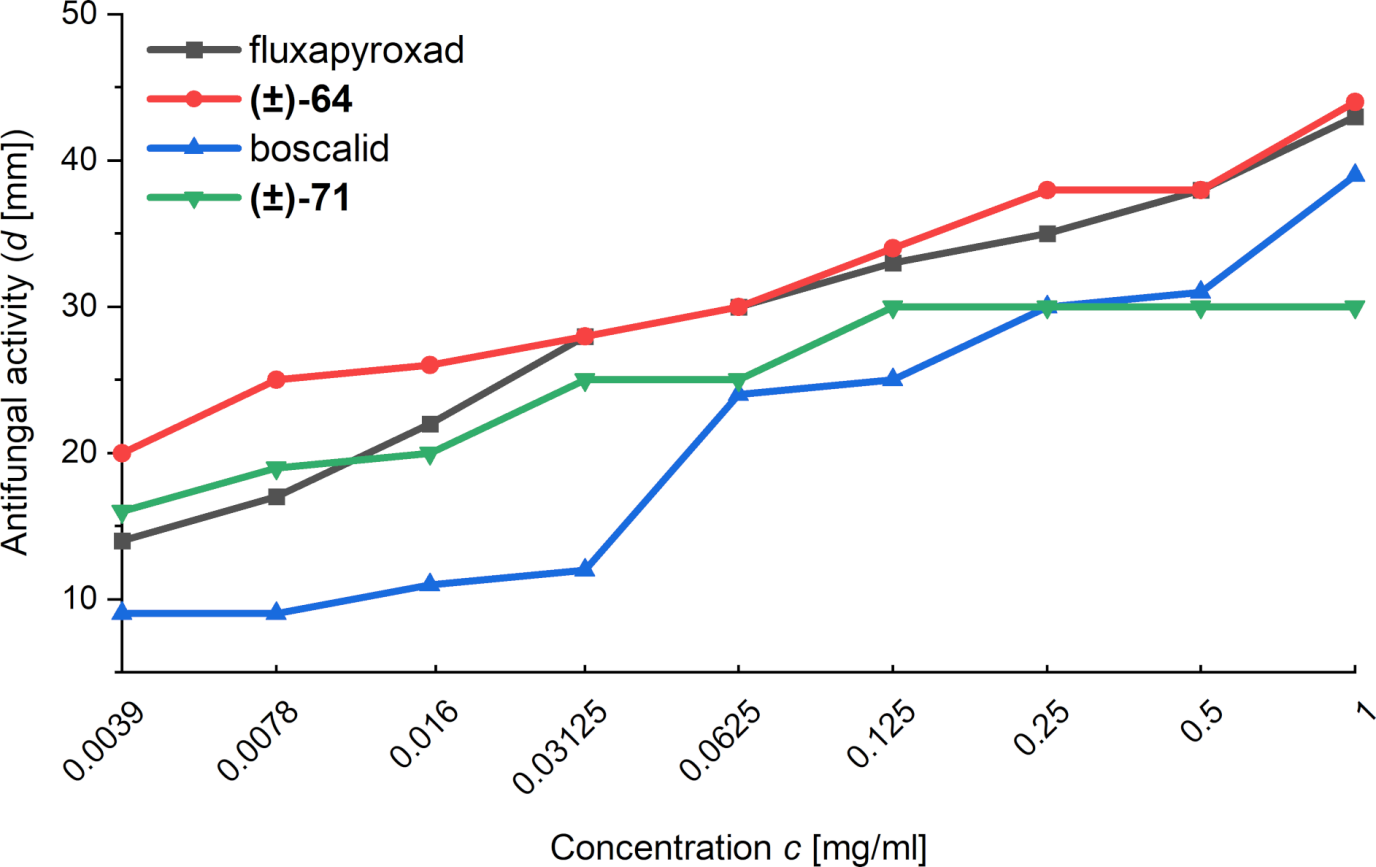
Antifungal activity of fluxapyroxad, its 1,5-BCH bioisostere **(±)-64**, boscalid and its bioisostere 1,5-BCH **(±)-71** against *Fusarium oxysporum* in dependence on the concentration of the fungicide [45].

Importantly, Mykhailiuk and co-workers also compared the antifungal activity of fluxapyroxad, boscalid and the racemic mixtures of bioisosteres 1,5-BCHs **(±)-64** and **(±)-71**. Their analysis showed that all compounds display similar antifungal activity over a wide range of concentrations, validating the proposition that 1,5-BCHs can function as bioisosteres of *ortho*-benzenes (Figure 9) [45].

#### 1,5-Disubstituted 3-oxabicyclo[2.1.1]hexanes

**Figure 10:**
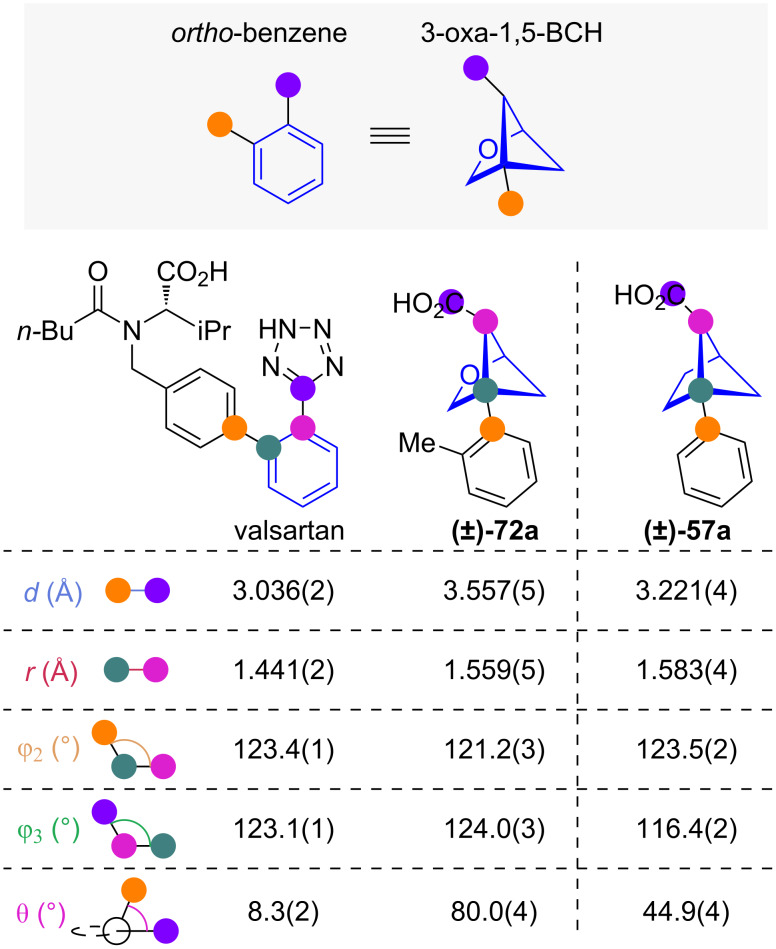
1,5-Disubstituted 3-oxabicylco[2.1.1]hexanes as isosteres for *ortho*-benzenes: Comparison of exit vector parameters for valsartan and 3-oxa-1,5-BCH **(±)-72a** and 1,5-BCH **(±)-57a** obtained from X-ray crystallography as reported by Mykhailiuk and co-workers [42,45].

To further increase the water solubility of 1,5-BCHs, Mykhailiuk and co-workers proposed 1,5-disubsituted-3-oxabicyclo[2.1.1]hexanes (3-oxa-1,5-BCHs) as isosteres for *ortho*-benzenes, introducing an oxygen atom into the hydrocarbon scaffold (Figure 10) [45]. Exit vector analysis reveals that the substituent distance *d* and distance between scaffold carbons *r* is slightly larger in 3-oxa-1,5-BCHs than in both *ortho*-benzenes and 1,5-BCHs [42]. The substituent scaffold angles φ_2_ and φ_3_ are on average actually closer to those of *ortho*-benzenes than for 1,5-BCHs. However, the dihedral angle θ of 3-oxa-1,5-BCHs of roughly 80° is even larger than in 1,5-BCHs and is a significant deviation from *ortho*-benzenes.

**Scheme 6:**
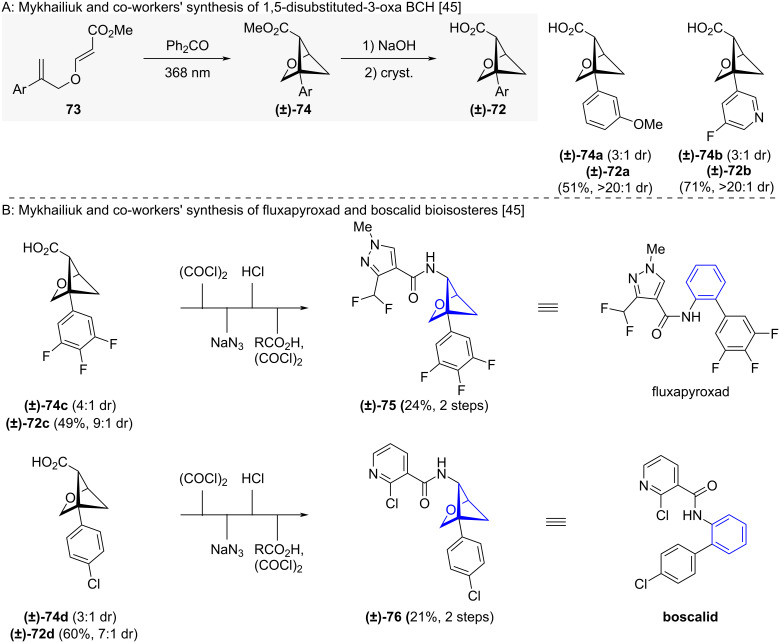
Synthesis of 1,5-disubstituted 3-oxabicyclo[2.1.1]hexanes as isosteres for *ortho*-benzenes via intramolecular crossed cycloaddition. A: Mykhailiuk and co-workers’ synthesis and representative substrate scope of 3-oxa-1,5-BCHs [45]. B: Mykhailiuk and co-workers’ synthesis of fluxapyroxad and boscalid bioisosteres as exemplary 3-oxa-1,5-BCH derivatizations [45].

Like their hydrocarbon 1,5-BCH analogues, 3-oxa-1,5-BCHs **(±)-74** can be accessed via an intramolecular [2 + 2] cycloaddition, and saponified to acids **(±)-72** (Scheme 6A) [45]. Chemical transformation of **(±)-72c** and **(±)-72d** led to bioisosteres **(±)-75** and **(±)-76** of fluxapyroxad and boscalid (Scheme 6B) [45].

**Figure 11:**
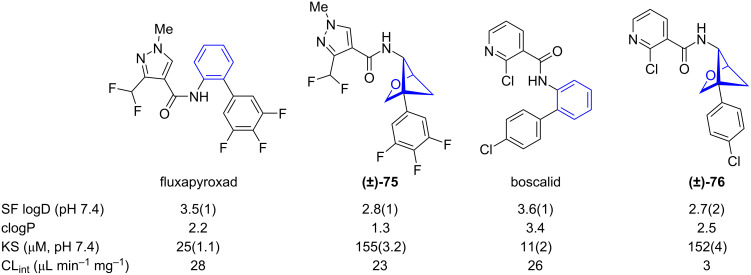
Comparison of physicochemical data of fluxapyroxad and boscalid and their 3-oxa-1,5-BCH bioisosteres **(±)-75** and **(±)-76** as reported by Mykhailiuk and co-workers [45]. Shake flask distribution coefficient (SF logD), calculated partition coefficient (clogP), kinetic aqueous solubility (KS), intrinsic clearance rate in human liver microsomes (CL_int_).

Comparison of the physicochemical data measured for fluxapyroxad and boscalid and their 3-oxa-1,5-BCH bioisosteres showed that bioisosteric replacement generally reduced the experimental distribution coefficient (SF logD) and the calculated partition coefficient (clogP) as well as increased the aqueous solubility (KS) (Figure 11) [45]. The intrinsic clearance in human liver microsomes (CL_int_) decreased upon bioisosteric replacement indicating increased metabolic stability.

**Figure 12:**
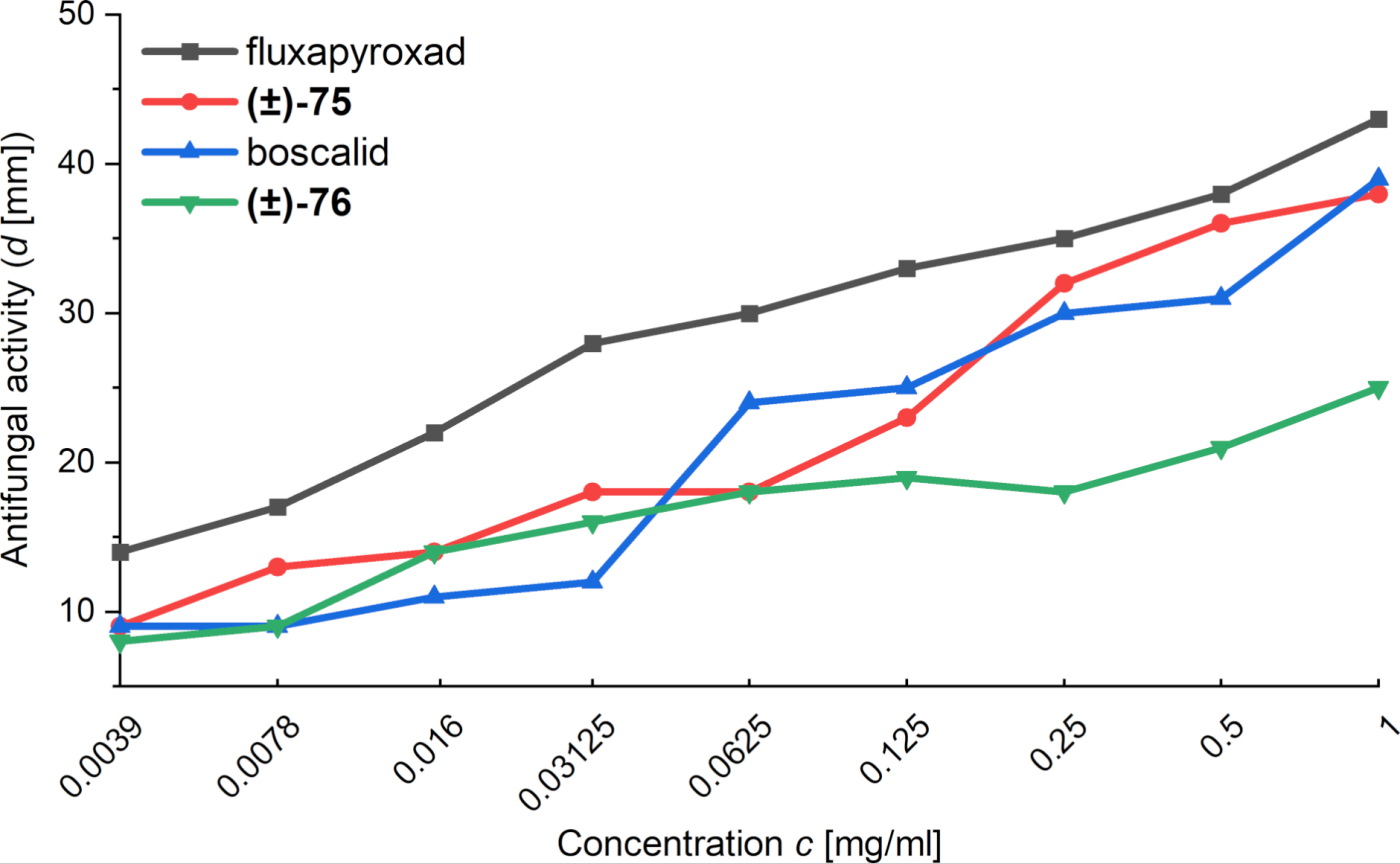
Antifungal activity of fluxapyroxad and boscalid and their 3-oxa-1,5-BCH bioisosteres **(±)-75** and **(±)-76** against *Fusarium oxysporum* [45].

In addition to the above physicochemical data, Mykhailiuk and co-workers assessed the antifungal activity of the suggested fluxapyroxad and boscalid bioisosteres **(±)-75** and **(±)-76** (Figure 12) [45]. The collected data shows that the isosteres, while still biologically active, are overall less active than the parent compounds over a wide range of concentrations. Nevertheless, the data indicates that the 3-oxa-1,5-BCH scaffold can be used as a bioisostere of an *ortho*-benzene.

#### 1,2-Disubstituted bicyclo[3.1.1]heptanes

Apart from the previously discussed isosteric replacements based on the bicyclo[1.1.1]pentane or bicyclo[2.1.1]hexane scaffolds, differently substituted bicyclo[3.1.1]heptanes (BCHeps) have also been suggested as isosteric replacements for *ortho*- or *meta*-benzenes [14,27,46–47].

**Figure 13:**
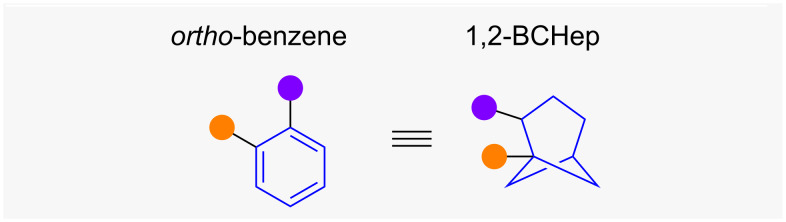
1,2-Disubstituted bicyclo[3.1.1]heptanes as isosteres of *ortho*-benzenes. Schematic representation of isosteric replacement of *ortho*-benzene with 1,2-BCHeps.

1,2-Disubstituted BCHeps (1,2-BCHeps) have been proposed as isosteres of *ortho*-benzenes [46], but no exit vector comparison has thus far been reported in the literature (Figure 13). Judgement of the geometrical likeness of 1,2-BCHeps and *ortho*-benzene therefore remains difficult. It can however be assumed that the dihedral angle θ of 1,2-BCHeps is once more significantly larger than the one of an equivalently substituted *ortho*-benzene.

**Scheme 7:**
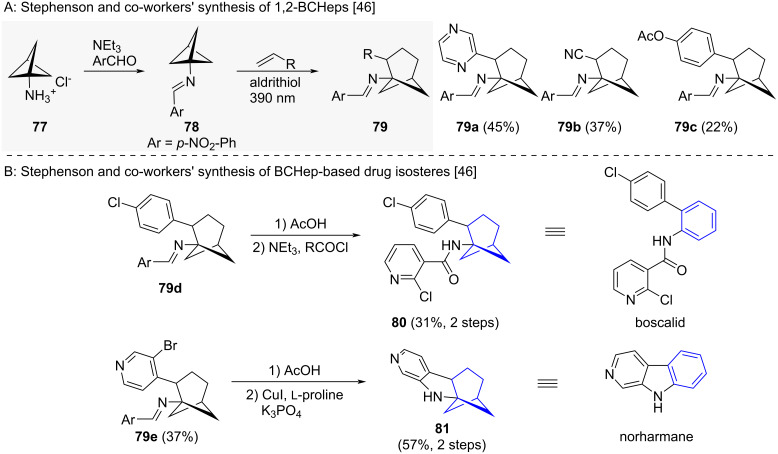
Synthesis of 1,2-disubstituted bicyclo[3.1.1]heptanes as isosteres for *ortho*-benzenes via alkene insertion. A: Stephenson and co-workers’ synthesis of 1,2-BCHeps as isosteres for *ortho*-substituted benzene and selected examples of the reported substrate scope [46]. B: Stephenson and co-workers’ synthesis of drug isosteres as exemplary derivatization reactions of 1,2-BCHeps [46].

Stephenson and co-workers accessed 1,2-BCHeps **79a–c** by insertion of alkenes into BCPs **78**, and proposed the 1,2-BCHeps as isosteres of *ortho*-benzenes (Scheme 7A) [46]. The reaction proceeded via homolytic cleavage of a C–C bond adjacent to the imine functionality and stepwise alkene addition and cyclisation. Styrenyl (to **79a,c–e**), acrylonitrile (to **79b**) as well as acrylate-based alkenes could be inserted under the reaction conditions. The authors were then able to apply their method to the synthesis of isosteres of both boscalid (isostere = **80**) and norharmane (isostere = **81**) (Scheme 7B) [46].

As with the exit vector parameters, there is currently no reported comparative physicochemical or biological data for 1,2-BCHeps with their equivalent *ortho*-benzene compounds.

#### 1,2-Disubstituted stellanes

**Figure 14:**
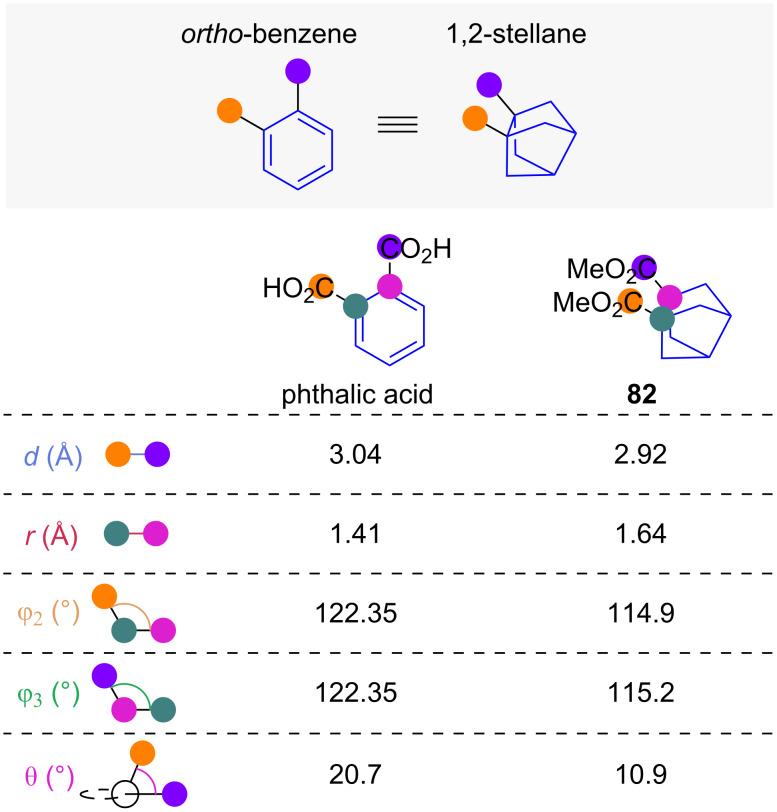
1,2-Disubstituted stellanes as *ortho*-benzene isosteres: Comparison of selected exit vector parameters in *ortho*-benzene phthalic acid [49] and 1,2-stellane **82** [48].

1,2-Disubstituted stellanes (1,2-stellanes) are an unusual scaffold that were recently proposed as *ortho-*benzene isosteres (Figure 14) [48]. Comparison of selected exit vector parameters obtained from reported crystal structures [48–49] indicates high geometric agreement between the stellane and benzene cores. Substituent distance *d*, scaffold carbon distance *r*, and the substituent scaffold angles φ_2_ and φ_3_ of stellane **82** all closely resemble those found in phthalic acid. While geometric consideration suggests that the dihedral angles θ of 1,2-stellanes should closely resemble the ones found in *ortho*-benzenes this is not the case in this comparison as the unusually high angle of 20.7° of phthalic acid is larger than the substituent dihedral angle θ of 10.9° of stellane **82**.

**Scheme 8:**
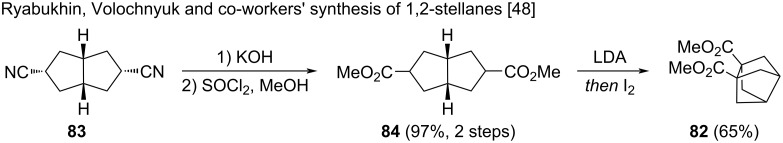
Synthesis of 1,2-disubstituted stellanes as isosteres for *ortho*-benzenes reported by Ryabukhin, Volochnyuk and co-workers [48].

The synthesis of 1,2-stellanes was originally reported in 1996 by Camps and co-workers [50]. Recently, Ryabukhin, Volochnyuk and co-workers modified Camps' synthetic protocol to access dimethyl stellane-1,5-dicarboxylate (**82**) on multigram scale (Scheme 8) [48]. As in Camps' synthesis, their approach starts from dinitrile **83**. However, while Camps and co-workers directly accessed the stellane scaffold from **83**, Ryabukhin, Volochnyuk and co-workers first transformed the nitrile groups into esters before forming stellane core **82**.

While the geometric properties of 1,2-stellanes indicate the scaffold to be a good geometric isostere of *ortho*-benzene, further research is required to increase the number of 1,2-stellanes with different functional groups and to evaluate the physiochemical and biological properties of these compounds.

#### 1,2-Disubstituted cubane

**Figure 15:**
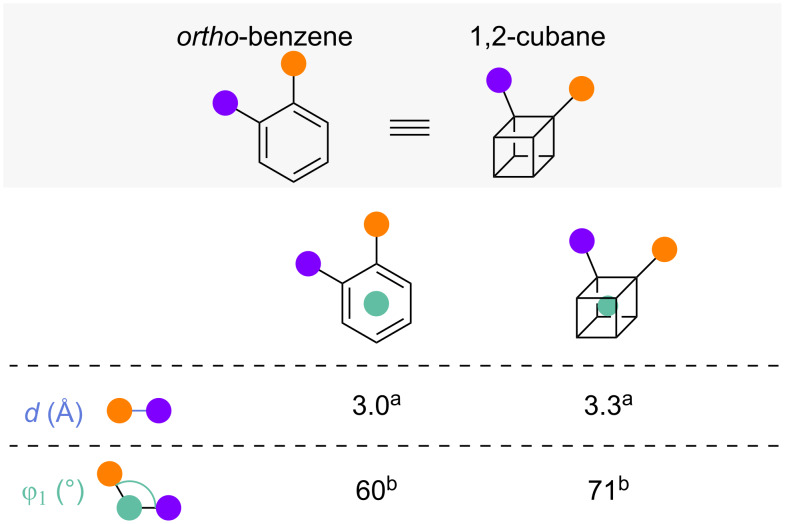
1,2-Disubstituted cubanes as *ortho*-benzene isosteres: Comparison of substituent distances and angles in *ortho*-benzene and 1,2-cubane as representative exit vector parameters. ^a^Reported by Mykhailiuk [14]. ^b^Reported by MacMillan and co-workers [51].

The final *ortho*-benzene isostere which will be discussed is 1,2-disubstituted cubane (1,2-cubane) [14,51]. No complete set of exit vector parameters of 1,2-cubanes is available in literature but substituent distances and angles in 1,2-cubanes have been reported and show close similarity to the corresponding *ortho*-benzenes (Figure 15) [14,51]. Even though no value for the dihedral angle θ of disubstituted cubane has been reported, it is likely that the values of θ are approximately equivalent, given the forced coplanarity of the substituents in both cubane and benzene [52].

**Scheme 9:**
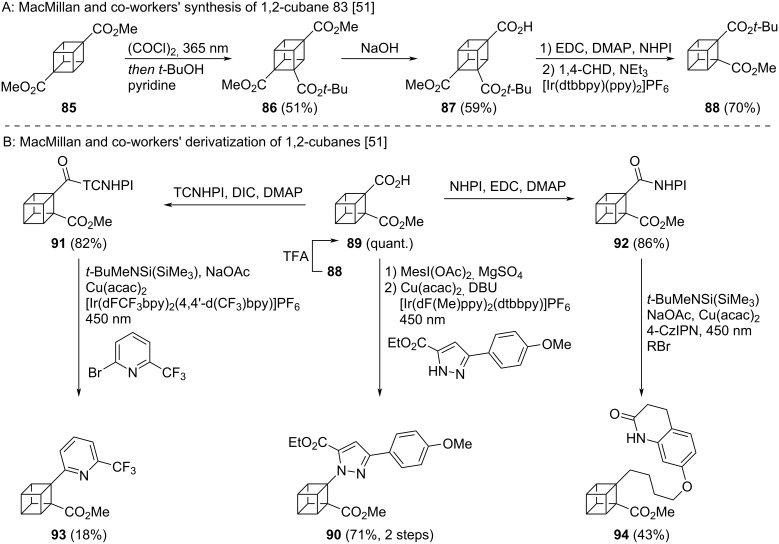
Synthesis of 1,2-disubsituted cubanes as isosteres for *ortho*-benzenes. A: Synthesis of 1,2-cubane developed by MacMillan and co-workers [51]. B: MacMillan and co-workers’ newly developed Cu-catalysed cross coupling reactions of 1,2-cubane [51]. NHPI = *N*-Hydroxyphthalimide, TCNHPI = *N*-hydroxytetrachlorophthalimide.

Routes to 1,4-cubanes have been available since the first syntheses of cubane derivatives by Eaton and co-workers [52–53]. Conversion of 1,4-cubanes to other disubstituted cubanes via a complex synthetic sequence has also been reported [54]. Recently, MacMillan and co-workers disclosed the development of a new synthetic route to diester 1,2-cubane **88** from dimethyl cubane-1,4-dicarboxylate (**85**) (Scheme 9A) [51]. 1,4-Cubane **85** is photochemically carboxylated to **86**, and selective saponification of the least hindered ester leads to **87**. Formation of a redox active ester followed by photocatalytical decarboxylation then yields 1,2-cubane **88**. This synthesis reduced the number of synthetic steps from eight, in the previously known patented synthesis from 2007 [54], to four. MacMillan and co-workers also developed a number of decarboxylative cross-coupling reactions to allow access to an even wider range of 1,2-cubanes (Scheme 9B) [51]. Partial deprotection of diester **88** led to acid **89** as a key intermediate and in situ activation of the acid as the hypervalent iodine complex enabled a photoredox decarboxylative amination to 1,2-cubane **90**. Alternatively, conversion of the acid moiety of **89** to redox active esters **91** and **92** enabled the metallaphotoredox arylation (to **93**) and alkylation (to **94**) of the cubane core. Comparative physicochemical or biological data for the 1,2-cubanes was not reported.

### Isosteres of *meta*-substituted benzenes

Many of the scaffolds previously discussed as potential bioisosteres for *ortho*-benzenes have, with different substitution patterns, also been suggested as potential bioisosteric replacements of *meta*-benzenes. In some cases, similar synthetic approaches may be adopted, but in many a new route is required to access the new substitution pattern.

#### 1,3-Disubstituted bicyclo[2.1.1]hexanes

**Figure 16:**
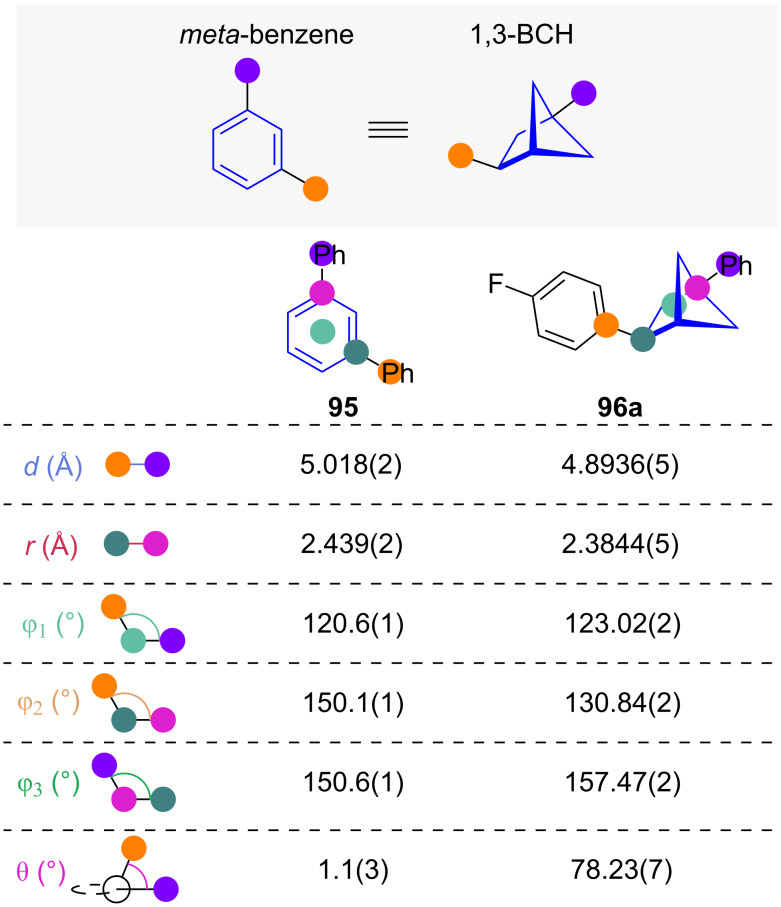
1,3-Disubstituted bicyclo[2.1.1]hexanes as isosteres of *meta*-benzenes: comparative exit vector parameters obtained from crystal structures reported by Walker and co-workers [34].

1,2-BCHs have been previously discussed as isosteres for *ortho*-benzenes (see Figure 4–Scheme 4). The related 1,3-disubstituted bicyclo[2.1.1]hexane (1,3-BCH) scaffolds have been suggested as isosteres for *meta*-benzenes (Figure 16) [14,34]. Exit vector analysis of 1,3-BCH **96a** shows that substituent distance *d*, scaffold carbon distance *r*, and substituent angle φ_1_ are remarkably similar to the aromatic counterpart. While the substituent–scaffold angles φ_2_ and φ_3_ show some deviation, the main difference is the 1,3-BCHs dihedral angle θ of 78° [34].

**Scheme 10:**
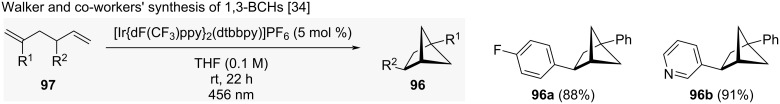
Synthesis of 1,3-disubstituted bicyclo[2.1.1]hexanes as isosteres for *meta*-benzenes reported by Walker and co-workers [34].

Recently, Walker and co-workers reported the synthesis of various polysubstituted BCHs, including 1,3-BCHs **96a** and **96b** via an intramolecular [2 + 2] cycloaddition from the corresponding diene **97** (Scheme 10) [34]. No comparative physicochemical or biological data was collected.

#### 1,4-Disubstituted bicyclo[2.1.1]hexanes

**Figure 17:**
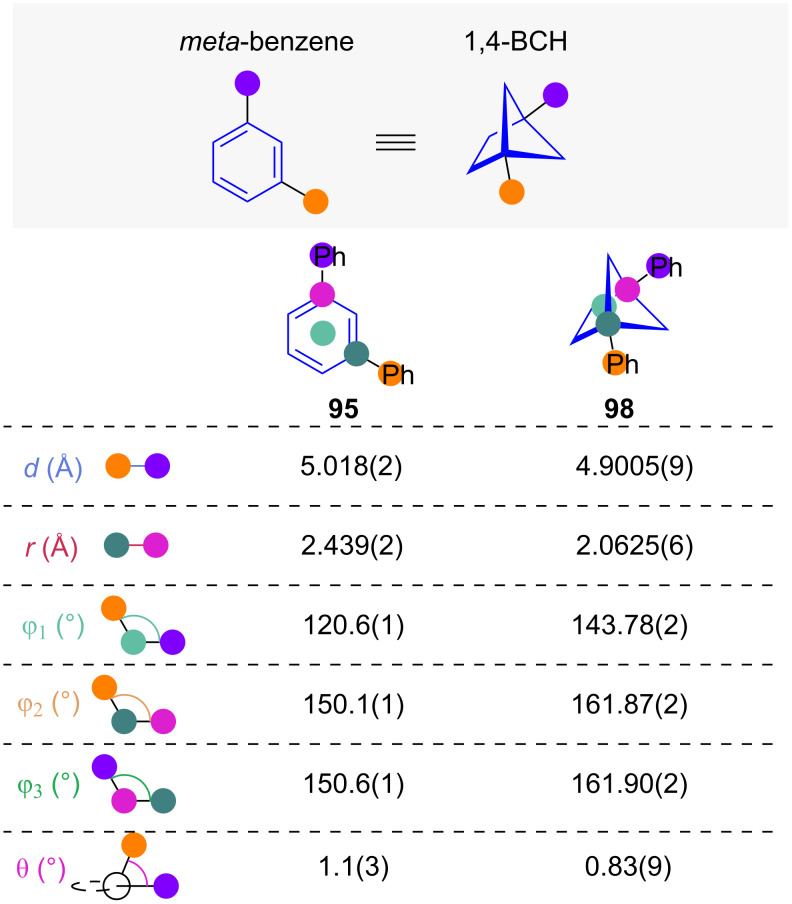
1,4-Disubstituted bicyclo[2.1.1]hexanes as isosteres of *meta*-benzenes: comparative exit vector parameters obtained from crystal structures reported by Walker and co-workers [34].

1,4-Disubsituted bicyclo[2.1.1]hexanes have been suggested as another BCH-based scaffold suggested for isosteric replacement of *meta*-benzenes [14]. Exit vector analysis of 1,4-BCH **98** and *m*-terphenyl (**95**) has been reported by Walker and co-workers (Figure 17) [34]. It shows that while the substituent distances *d* are remarkably similar, the carbon scaffold distance *r* of the 1,4-BCH is roughly 15% smaller. The substituent angle φ_1_ and the substituent scaffold angles φ_2_ and φ_3_ are all increased by isosteric replacement. Unlike for the 1,3-BCH, the dihedral angle θ for the 1,4-BCH is, with a value of 1°, near-identical to the aromatic equivalent.

**Scheme 11:**
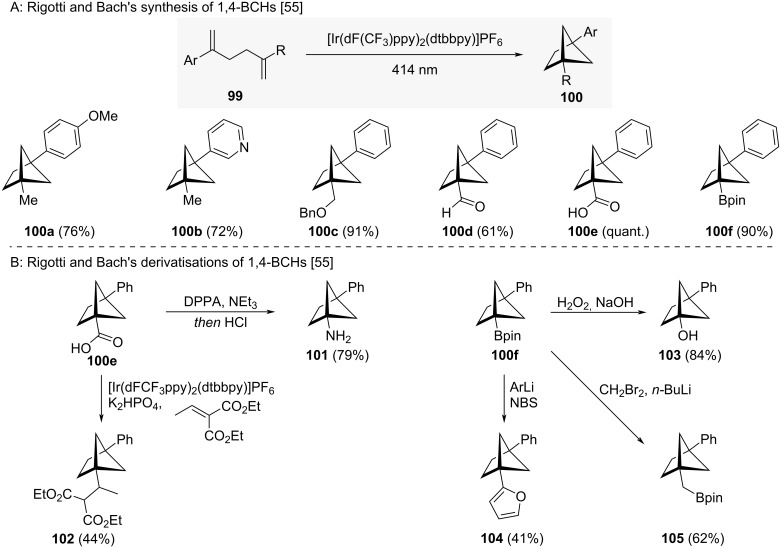
Synthesis of 1,4-disubstituted bicyclo[2.1.1}hexanes as isosteres for *ortho*-benzenes via intramolecular crossed cycloaddition. A: Rigotti and Bach’s synthesis of 1,4-BCHs [55]. B: Rigotti and Bach’s derivatizations of 1,4-BCHs [55]. DPPA = Diphenylphosphoryl azide.

Synthesis of 1,4-BCHs **100a–f** via intramolecular [2 + 2] cycloaddition of hexa-1,5-dienes was reported by Rigotti and Bach (Scheme 11A) [55]. These reactions require the presence of one styrenyl motif for efficient excitation. Derivatization of some of the compounds formed led to further useful 1,4-BCHs (Scheme 11B) [55]. From carboxylic acid **100e**, Curtius rearrangement led to amine **101** and a photoredox decarboxylative conjugate addition to diester **102**. From boronate ester **100f**, oxidative deborylation led to alcohol **103**, arylation led to furan **104** and Matteson homologation to boronate ester **105**.

Prior to Rigotti and Bach, a select few 1,4-BCHs had been synthesised by Qin and co-workers [41] and Blanchard [56]. Alternatively, Hartwig and co-workers developed a C–H borylation reaction to access bridgehead-borylated 1,4-BCHs from monosubstituted BCHs [57]. The synthesis of polysubstituted BCHs developed by Walker and co-workers has also been applied for the synthesis of 1,4-BCHs [34].

So far, no comparative physicochemical or biological data has been reported in the literature for these compounds.

#### 1,4-Disubstituted-2-oxabicyclo[2.1.1]hexanes

**Figure 18:**
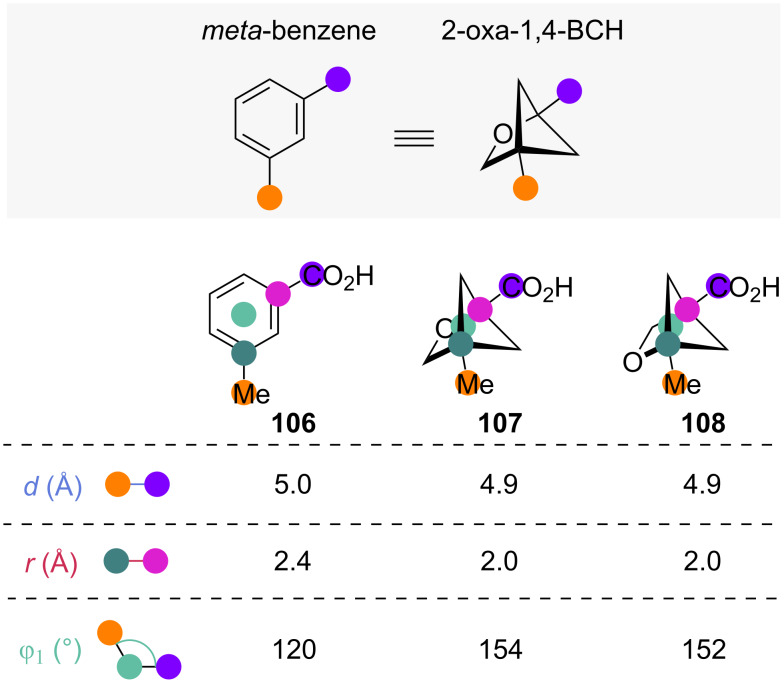
1,4-Disubstituted-2-oxabicyclo[2.1.1]hexanes as *meta*-benzene isosteres: comparison of selected exit vector parameters [58].

The introduction of 3-oxa-1,5-BCHs as more soluble and less lipophilic *ortho*-isosteres has previously been discussed (see Figure 10–Figure 12). The related 1,4-disubstituted-2-oxa-BCHs (2-oxa-1,4-BCHs) have also been investigated as *meta*-benzene isosteres (Figure 18) [58]. The exit vector analysis of 2-oxa-1,4-BCHs shows that the substituent distances *d* and scaffold carbon distances *r* are only slightly smaller than for the corresponding *meta*-benzenes. At the same time, the substituent angle φ_1_ is about 30° larger in 2-oxa-1,4-BCH.

**Scheme 12:**
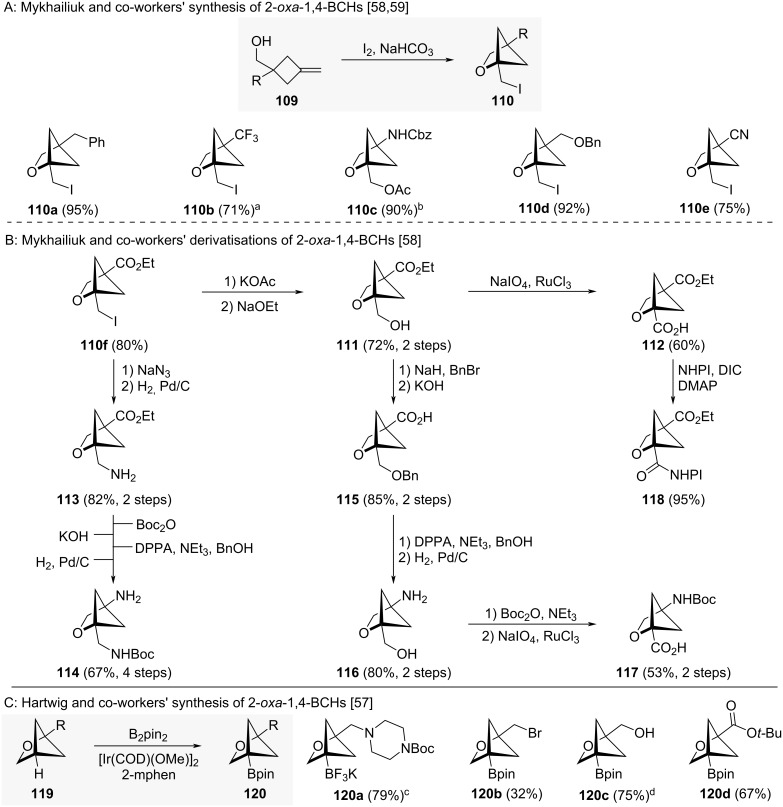
Synthesis of 1,4-disubstituted 2-oxabicyclo[2.1.1]hexanes as isosteres for *meta*-benzenes. A: Mykhailiuk and co-workers’ synthesis and representative substrate scope of 2-oxa-1,4-BCHs [58–59]. B: Synthesis of multifunctional 2-oxa-1,4-BCHs reported by Mykhailiuk and co-workers [58]. C: Hartwig and co-workers’ synthesis and substrate scope of 2-oxa-1,4-BCHs through C–H functionalisation [57]. ^a^As reported by Grygorenko and co-workers. ^b^The synthesised iodide was unstable and therefore treated with KOAc. ^c^Treated with KHF_2_ after completion of the reaction. ^d^In situ protection of the alcohol functionality with HBpin. NHPI = *N*-Hydroxyphthalimide, DPPA = diphenylphosphoryl azide, 2-mphen = 2-methylphenantroline.

Mykhailiuk and co-workers could synthesise the 2-oxa-1,4-BCHs **110a–f** in high yields by iodocyclisation of alcohols **109** (Scheme 12A) [58]. The formed products all contained a pendant primary iodide group and the synthesis was additionally shown to be tolerant of functional groups including protected amines (in **110c**), protected alcohols (in **110d**) and nitriles (in **110e**). Grygorenko and co-workers accessed fluorine-containing 2-oxa-1,4-BCH **110b** via Mykhailiuk’s synthesis [59]. 2-Oxa-1,4-BCH **110f**, which contained a bridgehead ester in addition to the pendant primary iodide, was found to be a versatile synthetic intermediate. Substitution of the primary iodide with acetate or azide led to alcohol **111** and amine **113,** respectively (Scheme 12B) [58]. Saponification of the ester moieties in these species followed by Curtius rearrangements then led to amines **114** and **116**. Non-natural amino acid derivatives **113** and **117** are intermediates prepared using these methods that could be of interest to medicinal chemistry. Alcohol **111** could also be oxidised to acid **112**, from which redox active ester **118** could be accessed.

An alternative approach to 2-oxa-1,4-BCHs involves the C–H-borylation of monosubstituted 2-oxa-BCHs developed by Hartwig and co-workers (Scheme 12C) [57]. Functional groups including halides (in **120b**), alcohols (in **120c**) and esters (in **120d**) are all tolerated under these conditions.

**Figure 19:**
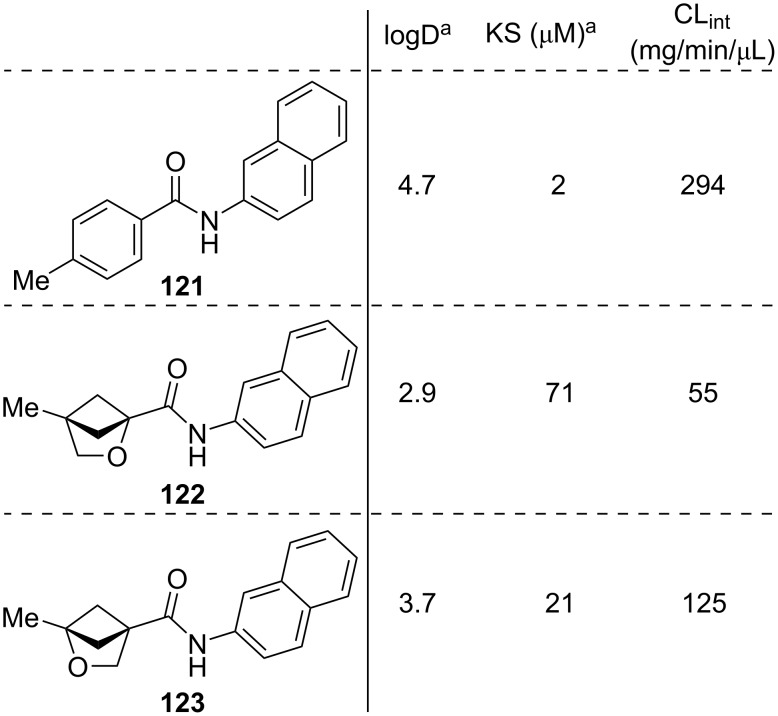
Comparative physicochemical data for 2- and 3-oxa-1,4-BCHs and *para*-substituted benzene equivalents as reported by Mykhailiuk and co-workers [58]. Experimental distribution coefficient (logD), kinetic aqueous solubility (KS) and intrinsic clearance in mouse liver microsomes (CL_int_). ^a^Measured at pH 7.4.

To the best of our knowledge, comparative physicochemical data for 2-oxa-1,4-BCHs and *meta*-benzenes has not been reported. However, Mykhailiuk and co-workers reported comparative physicochemical data for *para*-benzenes and both 2- (**122**) and 3-oxa-1,4-BCHs (**123**) (Figure 19) [58]. The experimental distribution coefficient (logD) of 2- and 3-oxa-1,4-BCHs **122** and **123** is reduced compared to equivalent *para*-benzene **121** while the kinetic aqueous solubility (KS) is increased. The intrinsic clearance rate in mouse liver microsomes (CL_int_) is also significantly reduced for both 2- and 3-oxa-1,4-BCHs compared with *para*-benzene **121** indicating higher metabolic stability.

#### 1,5-Disubstituted bicyclo[3.1.1]heptanes

**Figure 20:**
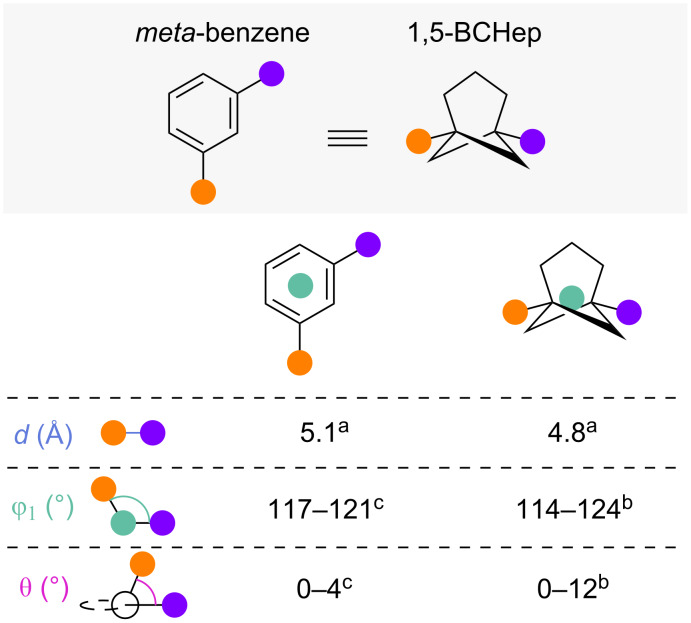
1,5-Disubstituted bicyclo[3.1.1]heptanes as isosteres of *meta*-benzenes: comparison of exit vector parameters [14,27]. ^a^Reported by Mykhailiuk and co-workers ^b^Obtained from single-crystal X-ray structures, reported by Anderson and co-workers ^c^Calculated on CPCM(THF)-B2PLYP-D3BJ/def2-TZVP level of theory, reported by Anderson and co-workers.

While only one investigation of BCHeps as potential *ortho*-benzene isosteres has been reported [46], their potential as *meta*-benzene isosteres is more widely investigated [14,27,47,60]. Comparison of selected exit vector parameters of 1,5-disubstituted bicyclo[3.1.1]heptanes (1,5-BCHeps) and *meta*-benzene was performed by Anderson and co-workers [27] and Mykhailiuk [14] (Figure 20). These analyses show that the substituent distance in 1,5-BCHeps is slightly smaller than in *meta*-benzene but the substituent and dihedral angles are remarkably similar.

**Scheme 13:**
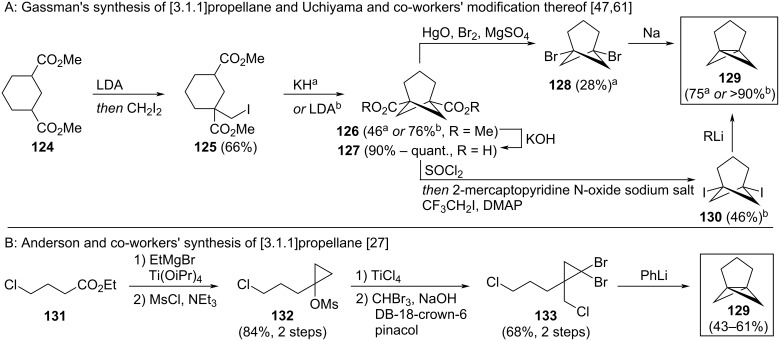
Synthesis of [3.1.1]propellane as a precursor for 1,5-disubsituted bicyclo[3.1.1]heptanes. A: ^a^Gassman’s original synthesis of [3.1.1]propellane [61] and ^b^Uchiyama’s modification thereof [47]. B: Anderson and co-workers’ synthesis of [3.1.1]propellane [27]. DB = Dibenzo.

1,5-BCHeps **126** and **127** were first reported by Gassman and Proehl [61] and by Wada [62]. The most common synthetic approach today is via the strain-release ring-opening of [3.1.1]propellane (**129**). Gassman reported the initial synthesis of [3.1.1]propellane (**129**) in 1980 [61], and this was recently optimised by Uchiyama (Scheme 13A) [47]. Cyclisation to the bridged structure **126** was achieved by enolate formation and intramolecular nucleophilic substitution of iodide diester **125**. A sequence of saponification (to **127**), decarboxylative halogenation (to **128** and **130**) and strain-inducing substitution then forms [3.1.1]propellane (**129**). Anderson and co-workers have reported a new synthesis of [3.1.1]propellane (**129**) based on the dibromocyclopropanation methodology typically used to access [1.1.1]propellanes (Scheme 13B) [27]. These new syntheses increased the overall yield of the [3.1.1]propellane synthesis from Gassman’s original 6% to 23% (Uchiyama) and up to 35% (Anderson) in 5 steps.

**Scheme 14:**
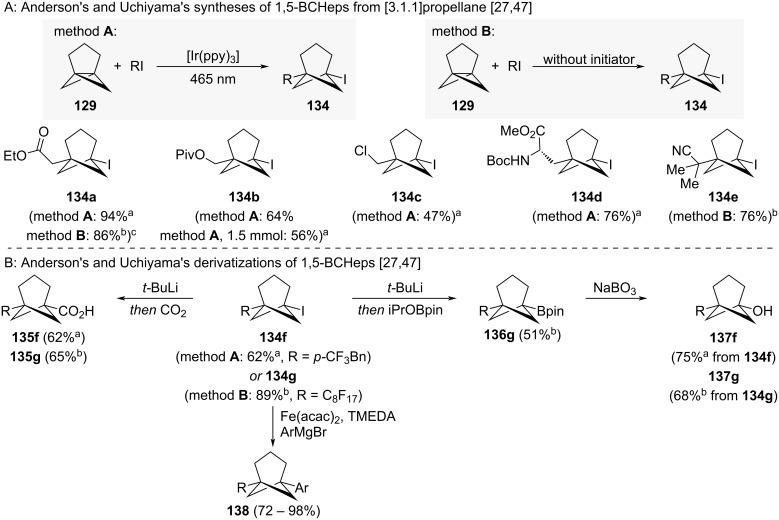
Synthesis of iodine-substituted 1,5-disubstituted bicyclo[3.1.1]heptanes as isosteres for *meta*-benzenes. A: Anderson’s and Uchiyama’s access of iodine-substituted 1,5-BCHeps [27,47]. B: Anderson’s and Uchiyama’s derivatization of iodine-substituted BCHeps [27,47]. ^a^Reported by Anderson and co-workers. ^b^Reported by Uchiyama and co-workers. ^c^Et_3_B can also be used as an initiator.

Iodine-substituted 1,5-BCHeps **134a–g** were shown to be accessible from [3.1.1]propellane via haloalkylation with alkyl iodides (Scheme 14A) [27,47]. This reaction can be performed either under photoredox catalysis conditions or without the need for an initiator, depending on the used alkyl iodide. For selected examples, the radical initiator Et_3_B could also be used. Activation by photoredox catalysis was developed by Anderson and co-workers and was shown to be the more versatile than initiator-free activation. Both initiator-free and Et_3_B-initiated reactions only tolerated electrophilic radicals (to **134a** and **134e**), while photoredox catalysis also tolerated electron-rich radicals (to **134b**). The synthesis of some 1,5-BCHeps, including **134b**, was also possible on mmol scale. Through derivatization of iodine-substituted 1,5-BCHeps **134f** and **134g**, an even larger number of 1,5-BCHeps were accessed (Scheme 14B) [27,47]. For example, lithium–halogen exchange was used to prepare acids **135f–g** and boronic ester **136g**. The latter could then be oxidised to the corresponding alcohols **137f–g**. The bridgehead iodine substituent could also be harnessed in iron-catalysed Kumada coupling reactions to furnish a larger number of arene-substituted 1,5-BCHeps **138**.

**Scheme 15:**
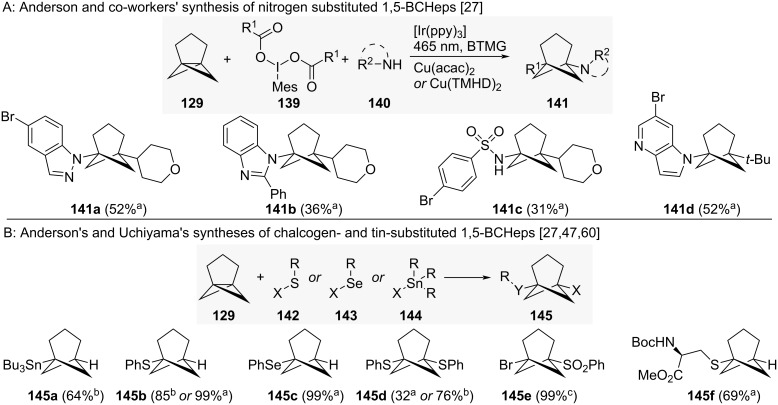
Synthesis of nitrogen-, chalcogen- and tin-substituted 1,5-disubstituted bicyclo[3.1.1]heptanes as isosteres for *meta*-benzenes. A: Anderson and co-workers’ synthesis of nitrogen-substituted 1,5-BCHeps [27]. B: Anderson and co-workers’ and Uchiyama and co-workers’ synthesis of chalcogen- and tin-substituted 1,5-BCHeps [27,47,60]. ^a^Reported by Anderson and co-workers. ^b^Reported by Uchiyama and co-workers. ^c^Reported by Anderson, Mykhailiuk and co-workers. BTMG = 2-*tert*-Butyl-1,1,3,3-tetramethylguanidine, TMHD = 2,2,6,6-tetramethyl-3,5-heptanedione.

Anderson and co-workers also reported access to nitrogen-substituted 1,5-BCHeps **141a–d** through a photoredox-catalysed aminoalkylation with amines **140** and iodonium dicarboxylates **139** (Scheme 15A) [27]. Both Anderson and Uchiyama also reported the synthesis of chalcogen- and tin-substituted 1,5-BCHeps **145a–f** from [3.1.1]propellane (Scheme 15B) [27,47,60].

**Figure 21:**
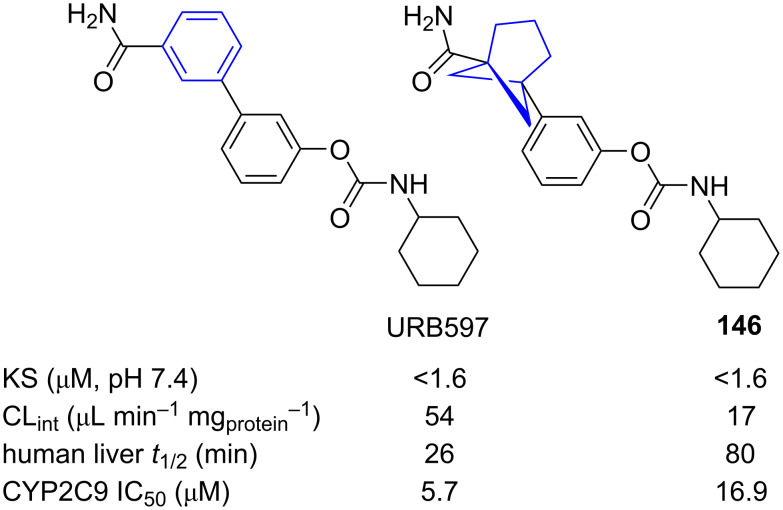
Comparative physicochemical data of URB597 and 1,5-BCHep isostere **146** [27]. Kinetic aqueous solubility (KS), intrinsic clearance in human liver microsomes (CL_int_), half-life in human liver microsomes (*t*_1/2_) and 50% inhibition concentration of CYP2C9 (IC_50_).

The ability of 1,5-BCHeps to act as bioisosteres of *meta*-benzenes was also studied by Anderson and co-workers. They reported the comparison of fatty acid amide hydrolase inhibitor URB597 with its 1,5-BCHep isostere **146** (Figure 21) [27]. They found that while replacement of one of the *meta*-benzenes does not change the aqueous solubility (KS), it does improve the intrinsic clearance rate in human liver microsomes (CL_int_) and the half-live in human liver microsomes (*t*_1/2_). Additionally, undesired target inhibition was reduced by isosteric replacement of *meta*-benzene with 1,5-BCHeps as shown by the 50% inhibition concentration of selected CYP450 enzymes (IC_50_).

#### *Cis-*2,6-disubstituted [2]-ladderanes

**Figure 22:**
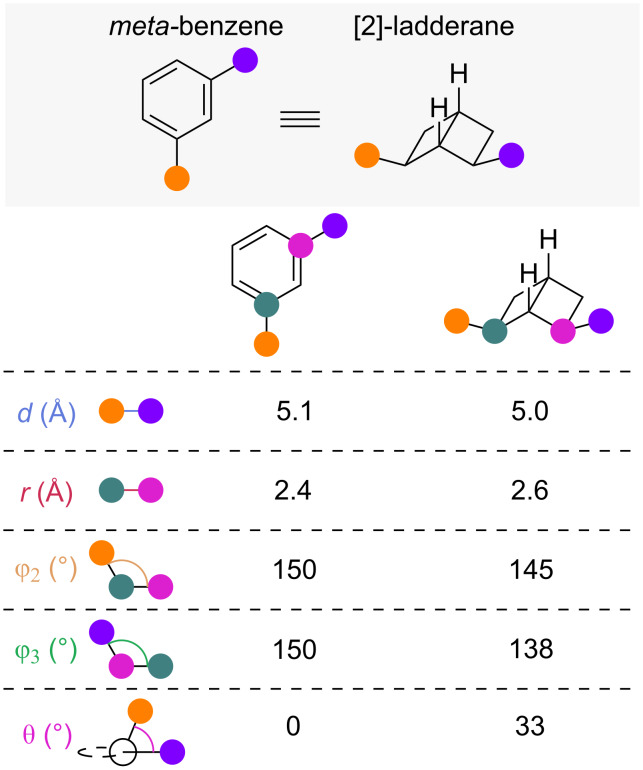
[2]-Ladderanes as isosteres of *meta*-benzenes: comparison of reported exit vector parameters [63].

Brown and co-workers recently proposed *cis-*2,6-disubstituted bicyclo[2.2.0]hexanes ([2]-ladderanes) as isosteric replacements for *meta*-benzenes. An exit vector analysis indicated that the substituent distance *d*, scaffold carbon distance *r*, and substituent scaffold angles φ_2_ and φ_3_ of [2]-ladderanes and *meta*-benzene are all very similar to those of *meta*-benzenes (Figure 22) [63]. However, as with many other proposed isosteres, the 30° dihedral angle θ of [2]-ladderanes is a significant deviation from planarity.

**Scheme 16:**
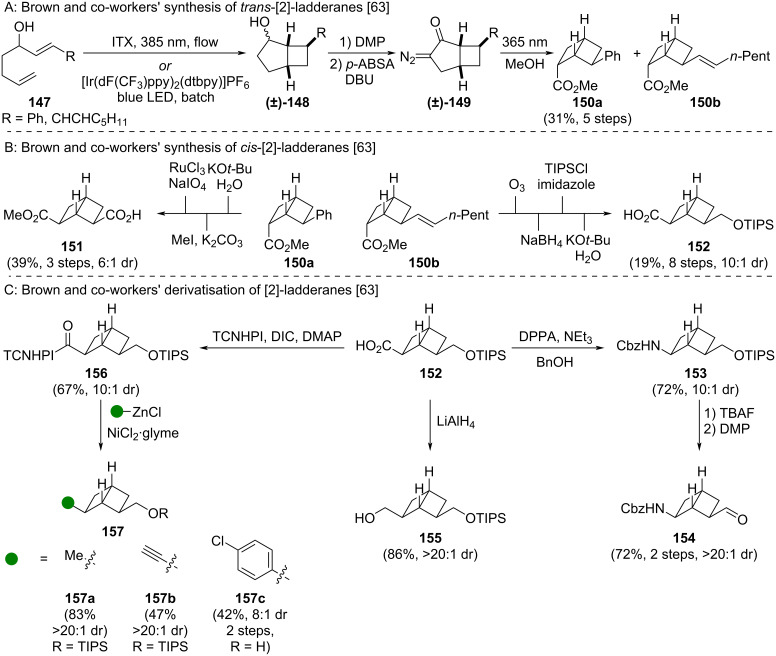
Synthesis of *cis*-2,6-disubstituted bicyclo[2.2.0]hexanes as isosteres for *meta*-benzenes. A: Brown and co-workers’ synthesis of *trans*-[2]-ladderanes **150a** and **150b** [63]. B: Transformation of *trans*-[2]-ladderanes into bifunctional *cis*-[2]-ladderanes **151** and **152** [63]. C: Selected derivatization reactions of *cis*-[2]-ladderanes [63]. ITX = Isopropylthioxanthone, *p*-ABSA = 4-acetamidobenzenesulfonyl azide, TCNHPI = *N*-hydroxytetrachlorophthalimide, DPPA = diphenylphosphoryl azide, DMP = Dess–Martin periodinane.

Brown and co-workers prepared the [2]-ladderanes via a multistep sequence from diene **147** including an intramolecular [2 + 2] cycloaddition (to **(±)-148**) and a Wolff rearrangement (to **(±)-150**) (Scheme 16A) [63]. This approach yielded predominantly the *endo* diastereomer of *trans*-[2]-ladderanes **150**. Isomerisation to the desired *cis*-[2]-ladderanes **150** was achieved through epimerisation of the ester functional group (Scheme 16B) [63]. Transformation of the phenyl group (of **150a**) or alkene group (of **150b**) into synthetically versatile acid **151** and protected alcohol **152** was achieved by standard chemical transformations. Additional bifunctional [2]-ladderanes were accessible by further synthetic manipulations (Scheme 16C) [63]. Acid **152** could be transformed into protected amine **153** by Curtius rearrangement and alcohol **155** through reduction. The [2]-ladderane scaffold was also shown to be tolerant of oxidising conditions in the transformation of alcohol **153** to aldehyde **154**. All of these transformations could be performed without reduction in diastereomeric ratio. Additionally, the authors showed that acid **152** can undergo nickel-catalysed decarboxylative cross coupling reactions via redox active ester **156** to afford alkyl, alkynyl, and aryl-substituted [2]-ladderanes **157a–c**.

**Figure 23:**
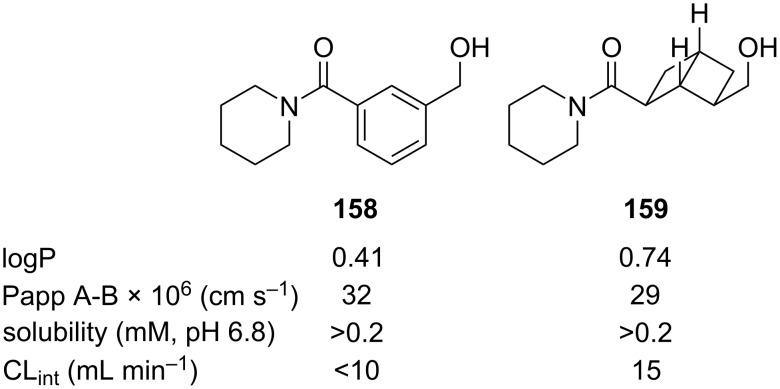
Comparative physicochemical data of *meta*-benzene **158** and [2]-ladderane isostere **159** [63]. Partition coefficient (logP), apparent permeability (Papp), equilibrium solubility and intrinsic clearance in rat liver microsomes (CL_int_).

Comparative physicochemical data of *meta*-benzene **158** and [2]-ladderane isostere **159** was also reported by Brown and co-workers (Figure 23) [63]. The comparison showed a modest increase in the partition coefficient (logP) and intrinsic clearance rate in rat liver microsomes (CL_int_), while apparent permeability (Papp) and equilibrium solubility are not significantly impacted by the isosteric replacement of the *meta*-benzene moiety.

#### 1,3-Disubstituted cubanes

**Figure 24:**
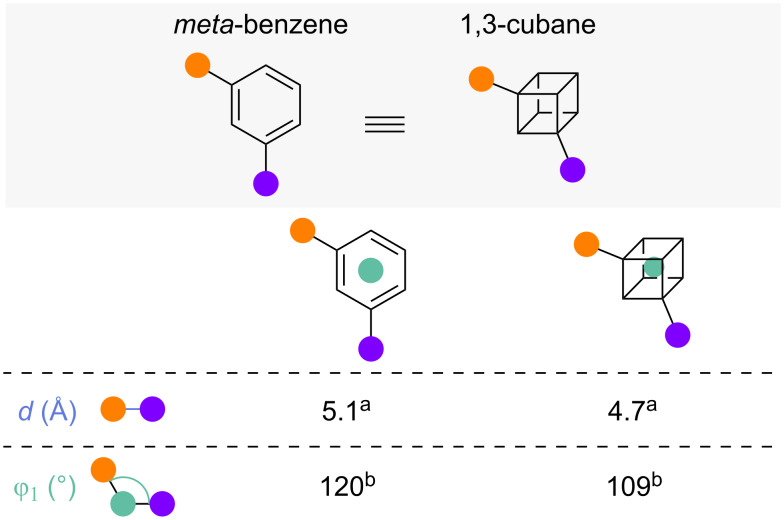
1,3-Disubstituted cubanes as isosteres of *meta*-benzenes: comparison of selected exit vector parameters [14,51]. ^a^Reported by Mykhailiuk. ^b^Reported by MacMillan and co-workers.

In addition to their improved synthesis of 1,2-cubanes as *ortho*-benzene isosteres, MacMillan and co-workers introduced viable routes to 1,3-cubanes as isosteres of *meta*-benzenes [51]. Exit vector analysis by Mykhailiuk [14] and MacMillan [51] showed that the substituent distance *d* and substituent angle φ_1_ are slightly smaller in 1,3-cubane than in *meta*-benzene (Figure 24). Even though no value for the dihedral angle θ of 1,3-cubane has been reported, it can be assumed, based on the forced coplanarity of the substituents in both disubstituted cubane and benzene [52], that the values of θ are approximately equivalent.

**Scheme 17:**
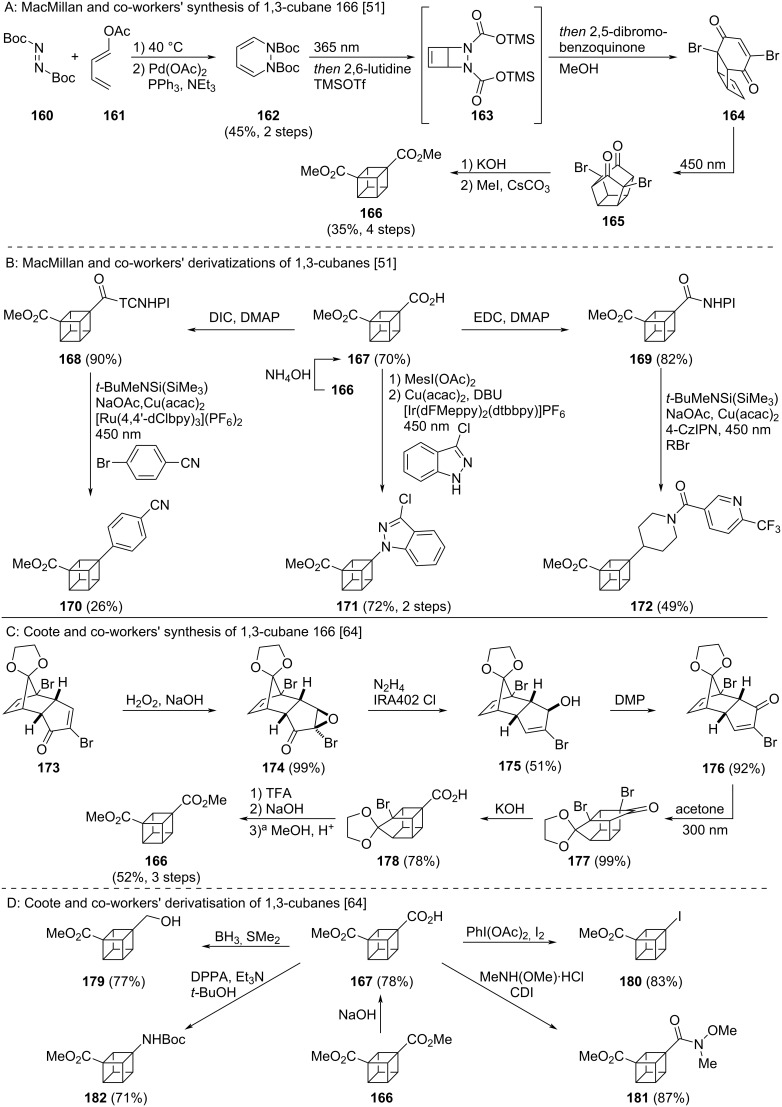
Synthesis of 1,3-disubsituted cubanes as isosteres for *meta*-benzenes. A: MacMillan and co-workers’ de novo synthesis of 1,3-cubanes [51]. B: Cross coupling reactions for derivatisations of 1,3-cubanes [51]. C: Coote and co-workers’ de novo synthesis of 1,3-cubanes [64]. D: Coote and co-workers’ derivatization of 1,3-cubanes [64]. ^a^Dowex 50W X8 resin was used as the proton source. NHPI = *N*-Hydroxyphthalimide, TCNHPI = *N*-hydroxytetrachlorophthalimide, DMP = Dess–Martin periodinane, CDI = carbonyldiimidazole, DPPA = diphenylphosphoryl azide.

1,3-Cubane diester **166** was the key synthetic target of MacMillan and co-workers, and was accessed over 8 steps from diene **161** (Scheme 17A) [51]. Diels–Alder reaction of diene **161** and di-*tert*-butyl azodicarboxylate (**160**) followed by palladium-assisted elimination of acetic acid gave diene **162**. A sequence of 4π-electrocyclisation and electrophilic transcarbamation (to **163**), deprotection, decarboxylation, oxidation, nitrogen extrusion (to cyclobutadiene) and Diels–Alder reaction yielded annulated tricycle **164**. Further intramolecular [2 + 2] cycloaddition formed cubane precursor **165**. From diketone **165**, 1,3-cubane **166** was obtained by Favorskii ring contraction followed by methylation of the intermediate diacid. As for the 1,2-cubanes, the authors were able to derivatise this general building block into a range of other 1,3-cubanes via metallophotoredox catalysis using acid **167** and redox active esters **168** and **169** (Scheme 17B) [51]. Arylation (to **170**), amination (to **171**) and alkylation (to **172**) were all possible.

An alternative pathway to 1,3-cubane **166** was reported by Coote and co-workers (Scheme 17C) [64]. Through their synthesis of enone **176**, used in Ueda’s synthesis of cubane **166** and previously only obtained as a side product of the Diels–Alder reaction forming enone **173** [65], they were able to access cubane **166** on gram scale. Synthesis of enone **176** was achieved by a Wharton transposition sequence [66]. The enone **173** was epoxidised yielding epoxide **174**, which could be converted into the allylic alcohol **175** by the Wharton reaction. Enone **176** could then be obtained by oxidation of alcohol **175** with Dess–Martin periodinane. Synthesis of cubane **166** was then achieved by [2 + 2] cycloaddition of enone **176** using acetone as the photosensitiser (to **177**), the first Favorskii ring contraction (to **178**), deketalisation, the second Favorskii ring contraction and esterification (to **166**) [64]. In addition to their synthesis of cubane **166**, Coote and co-workers also reported a number of bifunctional 1,3-cubanes accessible through functional group interconversion from acid ester cubane **167** (Scheme 17D) [64]. Reduction to the alcohol (to **179**) and iodination (to **180**) of the carboxylic acid motif, formation of the Weinreb amide **181** and Curtius rearrangement (to **182**) were all possible.

**Figure 25:**
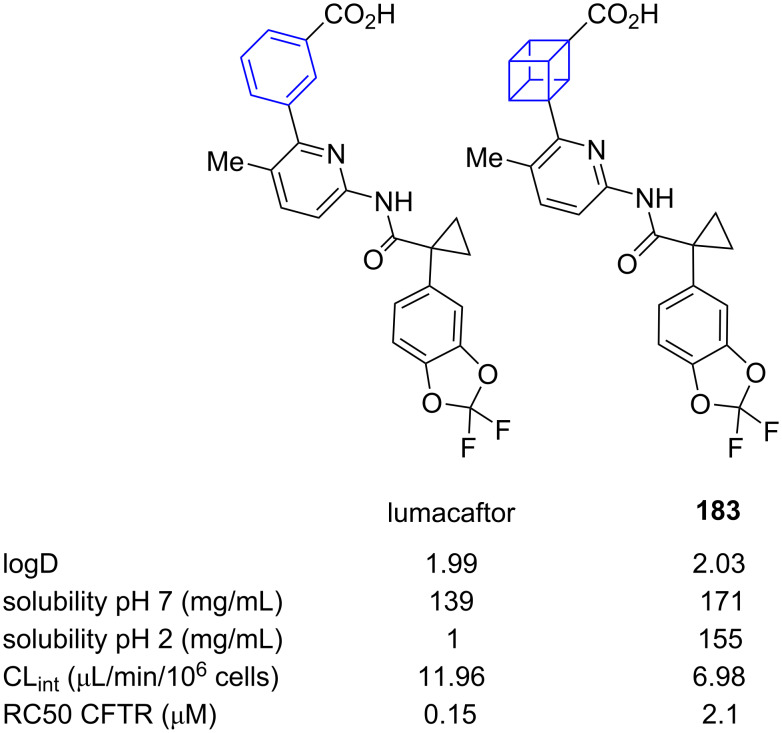
Comparative physicochemical data of lumacaftor and its 1,3-cubane bioisostere **183** [51]. Distribution coefficient (logD), intrinsic clearance in human liver microsomes (CL_int_), half-maximal rescue concentration (RC50 CFTR).

Comparative physicochemical and biological data for selected 1,3-cubanes and *meta*-benzene was reported by MacMillan and co-workers. They compared lumacaftor, one of the active compounds of the cystic fibrosis drug orkambi [67], to its 1,3-cubane bioisostere **183** (Figure 25) [51]. This comparison showed that while the distribution coefficient (logD) did not significantly change upon bioisosteric replacement, the solubility at neutral and low pH as well as the intrinsic clearance rate in human liver microsomes (CL_int_) improved significantly. The increase in solubility is particularly marked at low pH. A related observation was made by Poole and co-workers for quinoline-substituted 1,2,3-BCPs, where larger increases in the lipophilicity of the bioisosteric compound were also found at low pH [68]. One potential explanation for these observations is an increase in heterocycle basicity on bioisosteric switch of a neighbouring aromatic group. However, the drug-specific half-maximal rescue concentration (RC50 CFTR, lower indicates higher biological activity), indicative of the biological activity, increased by about one order of magnitude.

#### 1,3-Disubstituted cuneanes

**Figure 26:**
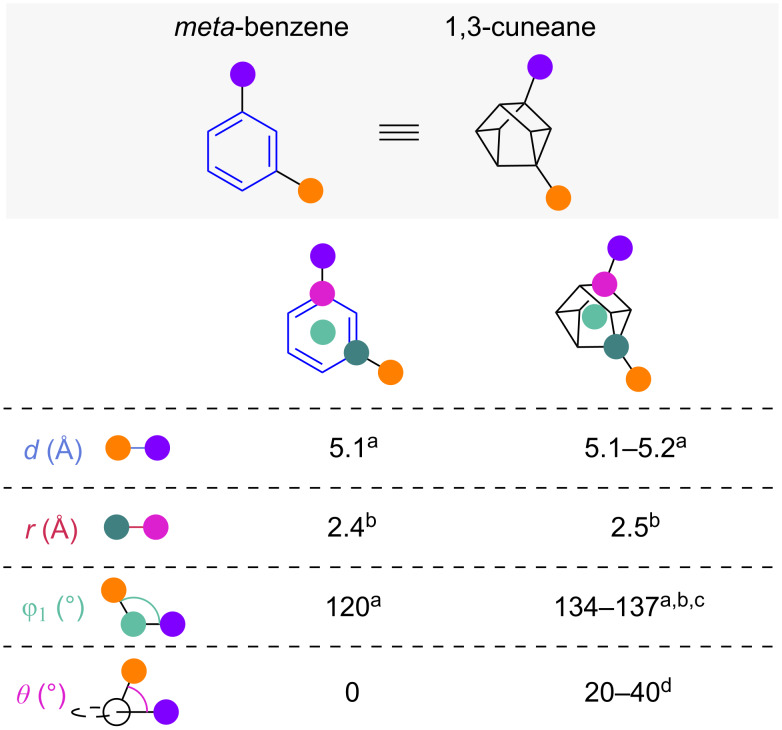
1,3-Disubstituted cuneanes as isosteres of *meta*-benzenes: comparison of selected exit vector parameters [70–72]. ^a^Reported by Lam and co-workers. ^b^Reported by Iwabuchi and co-workers. ^c^Reported by Stephenson and co-workers. ^d^Obtained from the crystal structures reported by Lam and co-workers and Stephenson and co-workers.

As a scaffold derived from cubanes, cuneanes have recently come into focus as potential bioisosteres. While the 1,3-disubstituted cuneanes (1,3-cuneanes) have been described as early as 1970 by Eaton and co-workers [69] their selective synthesis had not been studied until recently [70]. Selected exit vector parameters showed that substituent distance *d* and scaffold carbon distance *r* of 1,3-cuneanes closely resemble the distances found at *meta*-benzene (Figure 26) [70–72]. While the substituent angle φ_1_ of 1,3-cuneanes is only slightly larger than in *meta*-benzene, the dihedral angle θ is significantly larger and strongly dependent on the specific substituents.

**Scheme 18:**
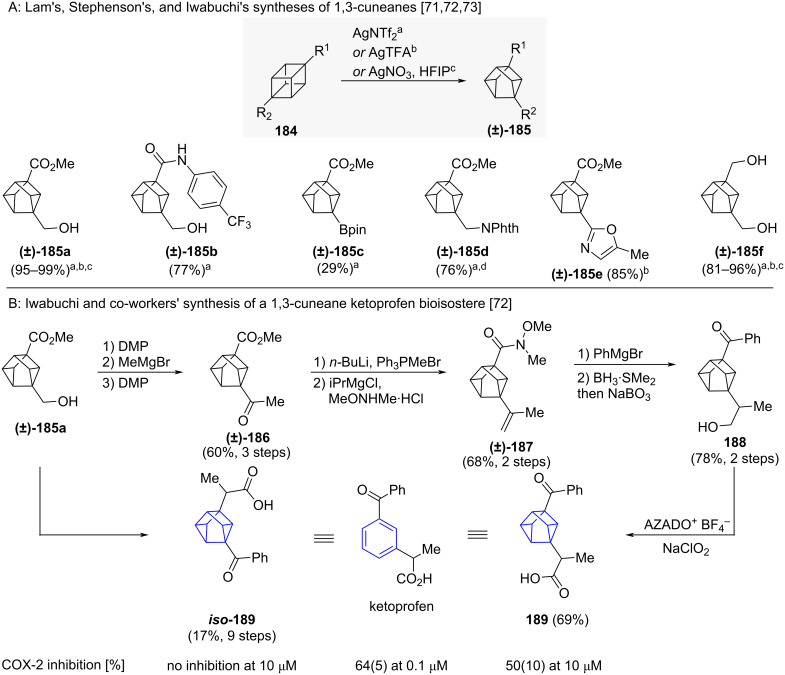
Synthesis of 1,3-cuneanes as isosteres of *meta*-benzene. A: Synthesis of 1,3-cuneanes reported by Lam and co-workers, Stephenson and co-workers and Iwabuchi and co-workers [70–72]. B: Synthesis of ketoprofen bioisostere **189** reported by Iwabuchi and co-workers [72]. ^a^Reported by Lam and co-workers. ^b^Reported by Stephenson and co-workers. ^c^Reported by Iwabuchi and co-workers. ^d^Yield corresponds to the isolated 3:1 mixture of 1,3-cuneane and 2,6-cuneane. HFIP = 1,1,1,3,3,3-Hexafluoro-2-propanol, AZADO^+^ = 2-azaadamantane *N*-oxyl.

1,3-Cuneanes can be obtained by metal-induced isomerisation of 1,4-cubanes [69]. Recent reports of their synthesis indicate that silver salts enable the isomerisation to occur efficiently (Scheme 18A) [70–72]. Experimental results and computational investigations by Stephenson and co-workers showed that the obtained ratio of 1,3-cunanes to 2,6-cuneanes is dependent on the cubane substituents [71]. Generally, at least one of the substituents needs to be electron donating to obtain the 1,3-cuneane selectively. The hydroxymethyl group (**185a, 185b, 185f**), a boronic acid pinacol ester (**185c**), a phthalimide-protected aminomethyl group (**185d**) and an oxazole (**185e**) were, among others, successful as electron-donating groups yielding 1,3-cuneanes. Iwabuchi and co-workers also found that the use of 1,1,1,3,3,3-hexafluoro-2-propanol (HFIP) as the solvent in the isomerisation reaction changes the obtained ratio in favour of the 1,3-cuneane when compared to their alternative solvent system of H_2_O/MeOH [72]. Iwabuchi and co-workers demonstrated the suitability of 1,3-cuneanes to undergo chemical transformations in their synthesis of ketoprofen bioisostere **189** (Scheme 18B) [72]. 1,3-Cuneane **186** bearing two electron-withdrawing groups was accessible by oxidation of 1,3-cuneane **185a**. The 1,3-cuneane scaffold was stable to Wittig olefination and Weinreb ester formation (to **187**). Reaction with a Grignard reagent, hydroboration and oxidation of the organoborane were also possible in high yields (to **188**). Similar to ketoprofen bioisostere **189**, its inversely substituted isomer ***iso*-189** was also accessible from **185a**. Iwabuchi and co-workers also investigated the biological activity of both **189** and ***iso*-189** in form of the inhibition of prostaglandin PGE_2_ synthesis by cyclooxygenase-2 (COX-2). Their investigation showed that while 1,3-cuneane **189** requires a 100-fold increase in concentration to reach inhibitory rates comparable to ketoprofen, its isomer ***iso*-189** is inactive against COX-2 (both **189** and ***iso*-189** were obtained and investigated as a mixture of four diastereomers).

**Figure 27:**
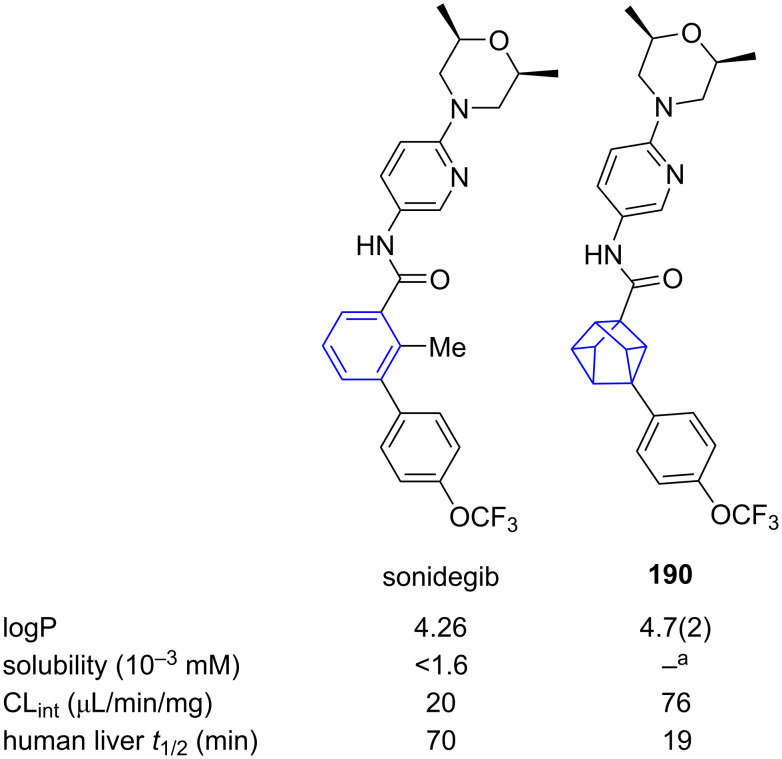
Comparative physicochemical data of sonidegib and its 1,3-cuneane isostere **190** [71]. ^a^Solubility was too low to be measured. Partition coefficient (logP), intrinsic clearance in human liver microsomes (CL_int_), half-life in human liver microsomes (*t*_1/2_).

Comparative physicochemical data of the anticancer drug sonidegib and its 1,3-cuneane isostere **190** was reported by Lam and co-workers (Figure 27) [71]. Unfortunately, in this case, isosteric replacement of *meta*-benzene with 1,3-cuneanes was found to not only have no significant effect on the partition coefficient (logP), but also significantly reduce both aqueous solubility and microsomal stability.

### Related polysubstituted scaffolds

**Figure 28:**
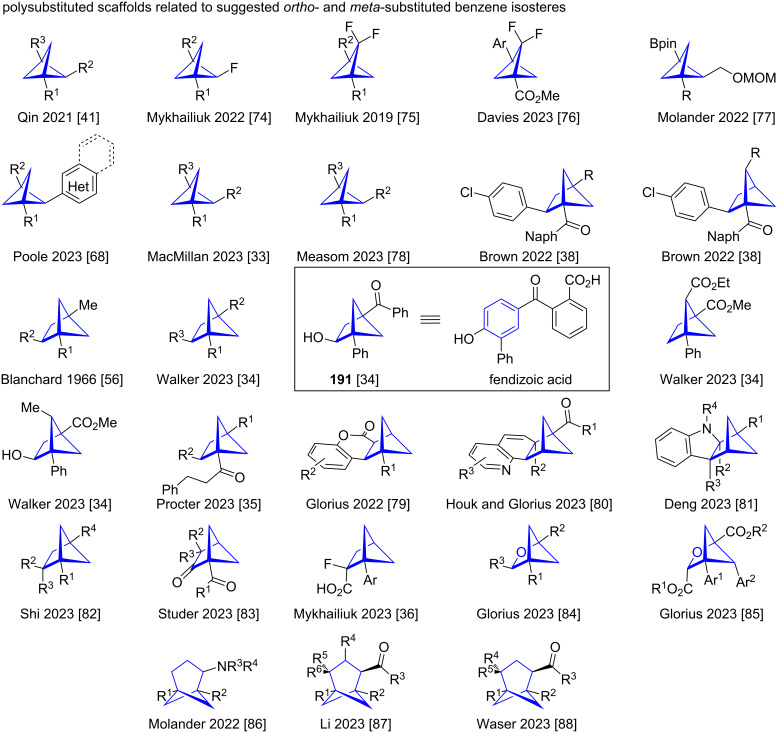
Exemplary polysubstituted scaffolds related to disubstituted scaffolds suggested as isosteres of *ortho*- or *meta*-benzene.

Apart from the discussed disubstituted scaffolds, a wide variety of different related tri- and polysubstituted scaffolds have been synthesised in the last few years, with the aim of providing more saturated and three-dimensional molecules for drug discovery. While many of them have not been explicitly suggested as potential isosteres for variously substituted benzenes, they are systems that widen accessible chemical space [73], have an increased number of stereogenic centres in accordance with Lovering's ‘Escape from Flatland’ approach [9], and could find application as alternatives to aromatic motifs in certain scenarios. Detailed discussion of their synthesis exceeds the goal of this review but a number of pertinent examples will be highlighted (Figure 28).

Bridge-substituted trisubstituted BCPs are potentially powerful building blocks offering unique substituent geometries for medicinal chemistry. A limited number of routes are available to these structures. Qin and co-workers employed an intramolecular coupling of sulfonylhydrazones and boronates to access 1,2,3-trisubstituted BCPs (1,2,3-BCPs). They also showed that their method can be extended to the synthesis of different bicyclic systems [41]. The synthesis of bridge mono-fluorinated 1,2,3,-BCPs by carbene insertion into bicyclobutane was reported in 2022 by Bychek and Mykhailiuk [74]. Previously the same group had also reported the synthesis of BCPs containing a difluorinated bridge by CF_2_ carbene insertion into bicyclobutanes [75]. More recently Davies and co-worker reported the one-pot synthesis of bridge difluorinated BCPs from α-allyldiazoacetates. In their reaction sequence, intramolecular cyclopropanation first forms the bicyclobutane which is then subjected to CF_2_ carbene insertion to yield the BCP [76]. Another useful pathway to 1,2,3-BCPs is strain release functionalisation as was shown by Molander and co-workers [77]. The synthesis of bridge heteroaryl 1,2,3-BCPs by a decarboxylative Minisci reaction was reported by Poole and co-workers [68]. The previously discussed C–H activation reported by MacMillan and co-workers (Scheme 2) was used to access a wide number of 1,2,3-BCPs [33]. A wide variety of 1,2,3-BCPs bearing substituents in the bridge position were reported by Measom and co-workers in their study on the lithium–halogen exchange of 2-bromo-1,2,3-BCPs [78].

Larger bridged bicyclic structures such as bicyclo[2.1.1]hexane offer multiple different exit vectors for substituents. It is therefore unsurprising that the number of methods seeking to access these has increased in recent times. In 2022 Brown and co-workers reported a strain release [2π + 2σ] cycloaddition induced by triplet energy transfer for the synthesis of 1,2,4-BCHs and 1,2,5-BCHs [38]. Some earlier examples of 1,2,4-trisubstituted BCHs were synthesised by Cairncross and Blanchard in 1966 by strain release [2π + 2σ] cycloaddition of bicyclobutane and alkenes [56]. Intramolecular crossed [2 + 2] cycloaddition frequently employed to access disubstituted BCHs is a useful tool in the synthesis of polysubstituted BCHs, as shown by Walker and co-workers in their synthesis of tri- and polysubstituted BCHs. These include 1,2,4-BCH **191**, which they described as a saturated analogue of fendizoic acid [34]. The previously discussed samarium-catalysed synthesis of BCHs (Scheme 3) reported by Procter and co-workers was also employed for the synthesis of 1,2,4-BCHs [35]. Heterocycle-substituted BCHs were accessed by [2π + 2σ] photocycloaddition by Glorius and co-workers [79] and Houk, Glorius and co-workers [80]. This reactivity was enabled by triplet energy transfer catalysis activating the heterocyclic substrate. Employing Lewis acid catalysis Deng and co-workers reported an alternative pathway to indole-derived BCHs. Polysubstituted BCHs were accessed by nucleophilic addition of the indole to the activated bicyclobutane followed by a Mannich cyclisation [81]. The synthesis of wide variety of tri- and tetrasubstituted BCHs was recently reported by Shi and co-workers [82]. They employed Hantzsch esters as catalysts in the [2π + 2σ] cycloaddition of alkenes and bicyclobutanes. The synthesis of bicyclohexyl ketones by formal (3 + 2) cycloaddition of bicyclobutane and ketenes was recently reported by Studer and co-workers [83]. Fluorine-substituted trisubstitued BCHs were accessed by Mykhailiuk and co-workers by photocatalysed intramolecular crossed [2 + 2] cycloaddition [36].

Glorius and co-workers also investigated the synthesis of polysubstituted 2-oxa-BCHs. Trisubstituted 2-oxa-BCHs were shown to be accessible by Lewis acid-catalysed cyclisation of bicyclobutanes with aldehydes [84] and polysubstituted 2-oxa-BCHs bearing more complex substitution patterns were synthesised by triplet energy transfer catalysis from benzoylformate esters and bicyclobutanes [85].

Reports of polysubstituted scaffolds larger than BCH are relatively rare. The synthesis of trisubstituted BCHeps has been reported by Molander and co-workers and Li and co-workers. Molander and co-workers accessed the BCHep scaffold by a photoinduced cycloaddition of bicyclobutanes and cyclopropylamines [86]. Li and co-workers recently reported the synthesis of polysubstituted BCHeps bearing a wide range of different substitution patterns. Their synthesis utilises boronyl radicals generated from diboron compounds by a pyridine cocatalyst to induce the [2σ + 2σ] cycloaddition of cyclopropanes and bicyclobutanes [87]. Similarly densely substituted BCHeps were synthesised by Waser and co-workers by photoinduced [2σ + 2σ] cycloaddition of bicyclobutanes and cyclopropanes [88].

## Conclusion

The last years have seen major progress in the development of new bioisosteres for *ortho*- and *meta*-benzenes, with a number of different options now available for both substrate classes. The range of synthetic approaches used also provides access to a variety of functional group handles on the bioisosteric scaffold, which should aid their adoption in drug discovery programmes. In some cases, the geometric similarity between bioisostere and benzene is less than ideal, particularly with regard to the dihedral angle between substituent exit vectors. Although in most cases the physicochemical properties of the isosteric compounds are nonetheless improved, with improved aqueous solubility, metabolic stability, and distribution often observed, the scarcity of data relating to biological activity for some isosteres makes a thorough assessment of their ability as bioisosteres, and the importance of the discrepancy in dihedral angle, difficult. More data in this direction would clearly be valuable to the field going forward. There is also clear potential in modifying the bioisosteric structure further, as exemplified by the inclusion of oxygen atoms in the BCH-derived bioisosteres [48,58]. The preparation of fluorinated scaffolds [34] potentially provides opportunities to further fine-tune lipophilicity of the drug candidate, as has already been explored for BCP-based *para*-benzene bioisosteres [75,89–90]. One final point to consider is the significant synthetic work required to prepare many of the discussed bioisosteres. Simple, scalable, routes to general building blocks, along the lines of the strain-release reactivity of [3.1.1]propellanes to BCHep *meta*-benzene bioisosteres, would represent a considerable contribution and aid adoption of these structures in the future.

## Data Availability

Data sharing is not applicable as no new data was generated or analyzed in this study.
